# In utero exposure to extreme heat increases neonatal mortality

**DOI:** 10.1093/pnasnexus/pgaf240

**Published:** 2025-08-19

**Authors:** Tenghui Wang, Jiafu An, Bin Chen, Chris John Webster, Peng Gong, Chen Lin

**Affiliations:** Department of Finance, Faculty of Business, Lingnan University, Hong Kong SAR 999077, China; Department of Real Estate and Construction, Faculty of Architecture, The University of Hong Kong, Hong Kong SAR 999077, China; Future Urbanity & Sustainable Environment (FUSE) Lab, Division of Landscape Architecture, Faculty of Architecture, The University of Hong Kong, Hong Kong SAR 999077, China; Institute for Climate and Carbon Neutrality, The University of Hong Kong, Hong Kong SAR 999077, China; Urban Environments and Human Health Lab, Faculty of Architecture, Hong Kong SAR 999077, China; Urban Systems Institute, The University of Hong Kong, Hong Kong SAR 999077, China; Department of Geography, The University of Hong Kong, Hong Kong SAR 999077, China; Department of Earth Sciences, The University of Hong Kong, Hong Kong SAR 999077, China; Faculty of Business and Economics, The University of Hong Kong, Hong Kong SAR 999077, China

## Abstract

Sub-Saharan Africa (SSA) has had the highest neonatal mortality rate in the world for the past few decades. In 2021, 27 infants died within the first month of life for every thousand live births in SSA, accounting for 54% of infant deaths (0–12 months) on the continent. Meanwhile, extreme heat events are occurring with increasingly higher frequency in this region due to climate change, worsening the living and health conditions for already vulnerable populations. Despite the prominence of high neonatal mortality rates and the frequent occurrence of extreme weather events in SSA, it remains unclear whether in-utero exposure to extreme heat is a significant contributing factor. Our research investigates this question using granular data on extreme heat and birth records from 33 African countries drawn from the Demographic and Health Surveys. It collects nationally representative, repeated cross-sectional surveys that assess reproductive and health behaviors across the developing countries every 5 years. Employing a measure of heat that simultaneously accounts for the impact of humidity, we show that a cumulative increase of 150 °C in extreme heat exposure during the 9-month pregnancy period is associated with two additional neonatal deaths per thousand live births. Mothers with lower economic status or limited education experience a more pronounced negative impact from exposure to extreme heat, likely due to reduced prenatal care. Our results are relevant to policymakers aiming to curb the negative impacts of climate change by better targeting the victims of extreme heat and developing effective adaptation strategies.

Significance Statement Our study addresses the critical issue of neonatal mortality in sub-Saharan Africa—a region burdened by high infant death rates and increasingly frequent extreme heat events due to climate change. Using detailed data from 33 African countries, we found that exposure to extreme heat and humidity during pregnancy significantly increased the risk of neonatal death, with rural, economically disadvantaged, and less educated mothers disproportionately affected. These findings underscore the urgent need for targeted interventions to protect vulnerable populations from the health impacts of climate change. By identifying specific groups at greater risk, our research provides valuable insights for policymakers seeking to develop effective strategies to mitigate the effects of extreme heat on maternal and infant health.

## Introduction

**Fig. 1.**
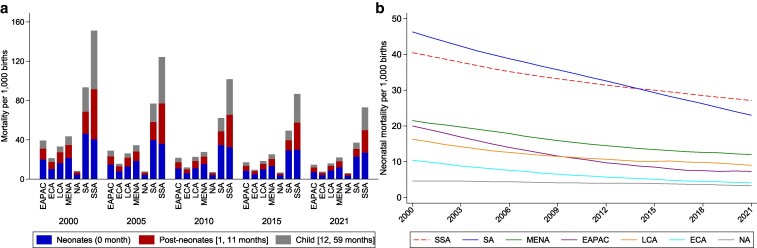
Mortality rates across continents. This figure shows the change in mortality rates across continents. a) Neonatal mortality, postneonatal mortality, and child mortality by continent per 1,000 births in 2000, 2005, 2010, 2015, and 2021. The sum of neonatal mortality and postneonatal mortality constitutes infant mortality (death before reaching the age of 12 months). Infant mortality plus child mortality equals under-5 mortality. b) The time-series pattern of neonatal mortality by continent from 2000 to 2021. SSA, sub-Saharan Africa, SA for South Asia, MENA for the Middle East and North Africa, EAPAC for East Asia & Pacific, LCA for Latin America and Caribbean, ECA for Europe and Central Asia, and NA for North America.

Since the turn of the millennium, sub-Saharan Africa (SSA) has witnessed a significant reduction in under-5 mortality rates (1), plummeting from 151 to 73 deaths per 1,000 live births, a testament to the substantial progress made in improving child survival rates in the region. However, neonatal mortality, deaths within the first month of life, remains a persistent challenge, disproportionately impacting overall child mortality risk in SSA. In 2021, neonatal deaths accounted for 37% of under-5 deaths and 54% of infant deaths, a marked increase from the year 2000. Despite improvements, SSA continues to bear the highest neonatal mortality rate globally. This trend contrasts with South Asia (SA), a comparable region in development levels, where neonatal mortality rates have also been high but have decreased at a faster rate since 2000 (see Fig. 1). The slower decline in SSA's neonatal mortality rates, despite significant reductions in under-5 mortality, underscores the critical need to search for underlying causes and tailor interventions toward the most vulnerable subpopulations (2).

The backdrop of these mortality trends is the increasingly severe climate conditions witnessed globally, with the summer of 2023 marking unprecedented extreme temperatures and persistent heatwaves. Around 30% of the global population now faces temperatures above the lethal threshold of at least 20 days annually (3, 4). Pregnant women, particularly in SSA, are at a heightened risk of adverse outcomes due to extreme heat, which can induce complications such as reduced placental blood flow and dehydration (5–8). These conditions are detrimental to fetal development, potentially increasing neonatal mortality rates (9–11). The vulnerability of SSA to climate change suggests a compelling link between rising frequency of extreme heat events and neonatal deaths. Given the established impact of environmental conditions on neonatal health, the persistent and escalating heat stress in SSA is likely to be a significant factor impeding progress in reducing neonatal mortality, highlighting the urgent need for targeted climate adaptation and healthcare strategies (12).

**Fig. 2.**
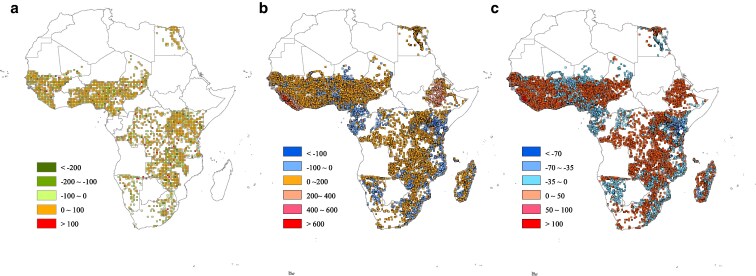
Changes of neonatal mortality and extreme heat exposure. The figure illustrates changes in neonatal mortality and extreme heat exposure in our research sample. Panel a) illustrates the differences in neonatal death rates between high- and low-heat exposure regions over time, while panels b) and c) show the differences in cumulative extreme heat and extreme heat days, respectively, over time.

In this study, we quantify the impacts of prenatal exposure to extreme heat on neonatal mortality rates across the African continent, highlighting potential policy intervention strategies. Using the Demographic and Health Surveys (DHS), a data source organized by ICF which collects nationally representative, repeated cross-sectional surveys that assess reproductive and health behaviors,^a^ we analyze extreme heat exposure for 883,623 birth records from 32,694 DHS survey clusters within 3,500 two-degree grid cells across 33 countries, covering births from October 2006 to June 2020, to provide a comprehensive overview of how in utero heat exposure affects neonatal mortality. Our analysis, detailed in Fig. S1 and Table S5, reveals a dual trend: a general decline in neonatal mortality rates, alongside an increase in the intensity of extreme heat experiences by 2020 compared with 2006, as illustrated in Fig. 2.

Despite global progress in reducing child mortality, neonatal deaths remain a pressing challenge in SSA. According to recent estimates, approximately three out of every 100 newborns in the region die within their first month of life. While 133 out of 200 countries have already achieved the Sustainable Development Goal (SDG) target on child survival, projections indicate that, without significant additional efforts, Africa will not meet this target until the end of the century. This persistent gap underscores the urgency of identifying the key drivers of neonatal mortality in the region. Our study contributes to this goal by providing robust empirical evidence on the role of in utero exposure to extreme heat—a rapidly intensifying risk factor under climate change. By highlighting both the magnitude and mechanisms of this relationship, our findings offer timely insights for designing targeted interventions to help accelerate progress toward the SDG child survival target in the African context.

Our study confronts two significant challenges in pinpointing the health implications tied to exposure to extreme temperatures. The first hurdle is the precise measurement of temperature exposure, now made possible through advancements in remote sensing technologies. Utilizing the Global Seamless 1 km Resolution Daily Land Surface Temperature Dataset (13), we achieve a high level of spatial and temporal resolution in our daily temperature data, allowing for an accurate mapping of data for linking heat exposure and neonatal mortality. The precision in data linkage enables us to estimate a causal link between prenatal exposure to extreme temperatures and rates of neonatal deaths in Africa.

The second hurdle involves mitigating the confounding effects of birth seasonality, which can influence both the timing of births and the associated health outcomes due to parental preferences for specific birth periods (14). Moreover, both birth seasonality and temperature fluctuations are linked to factors affecting fertility and parental characteristics, which may in turn impact infant health (15–17). To navigate these complexities and isolate the specific effects of prenatal heat exposure on neonatal mortality, our study design includes 2° grid cell-birth month, birth year, and DHS cluster fixed effects. This approach allows us to isolate the direct impact of extreme heat exposure by identifying heat effects within a region while controlling for time trends. As a result, we can account for and eliminate unobservable regional and temporal confounding factors.

Our empirical evidence shows that prenatal exposure to extreme heat increases neonatal mortality. When considering the type of residence, we found that the negative impacts are concentrated in rural areas, while urban regions showed no significant response to prenatal heat exposure. These findings highlight the urban–rural divide. By demonstrating that in utero exposure to extreme heat significantly raises neonatal mortality, our research contributes to the literature on the “fetal origins” hypothesis, which suggests that environmental, nutritional, and other mild shocks during pregnancy can adversely affect child health (18, 19). Our evidence underscores the urgent need for early-stage investments to mitigate these negative outcomes starting from the prenatal period. While the impact of heat exposure on fetal development is acknowledged, the precise physiological mechanisms remain largely unexplored (9). Our research indicates that reduced prenatal checkups and the increased prevalence of water-borne diseases—due to inadequate water and sanitation infrastructure—may play a significant role. These findings emphasize the urgent need to improve healthcare access and water safety standards.

Our analysis identifies key vulnerable groups in rural areas who are disproportionately affected by these challenges, particularly those from low-income backgrounds, households without electricity, and mothers with limited education. By highlighting the acute needs of these subpopulations, our findings advocate for targeted interventions, especially in resource-constrained rural settings. We propose several adaptive strategies to address these vulnerabilities, including the deployment of community health workers, investments in education and electricity infrastructure, alongside broader efforts toward economic development to reduce the urban–rural divide in the long term. Our research has significant implications for policymakers, emphasizing the need for coordinated actions to mitigate the adverse effects of global warming on maternal and child health in underdeveloped regions. Through such targeted initiatives, we aim to contribute to the global discourse on addressing climate inequality and fostering a healthier future for vulnerable populations across Africa.

### Extreme heat exposure during pregnancy increased neonatal mortality

The distinctive challenge posed by high temperatures, especially when combined with high humidity, is pronounced in Africa. Thermal comfort changes dramatically as temperatures rise, primarily because humans are endothermic and depend on the process of sweating for cooling (20). High humidity levels interfere with this natural cooling mechanism, significantly impairing the body's ability to manage heat stress (21–23). To factor this into a continental scale analysis of thermal stress across climatic conditions, our study employs the measurement of wet-bulb temperature. This metric integrates relative humidity with ambient temperature to more accurately reflect thermal comfort experienced by individuals.

Recognizing the cyclical nature of temperatures throughout the year and the existence of a human comfort zone, we adopted a novel approach by selecting specific temperature percentiles (75th, 85th, 90th, and 95th) within the same season over a 3-year period to establish temperature thresholds. By comparing daily temperatures against these thresholds, we identify and quantify abnormal heat exposure, focusing on the intensity of exposure (intensive margin) during the critical 9-month pregnancy period. Additionally, we incorporate an alternative measure of heat exposure by counting the number of days individuals are subjected to extreme high temperatures (extensive margin). This dual approach enables us to capture both the frequency and intensity of heat exposure, providing the first comprehensive understanding of prenatal heat stress across SSA and its potential impact on neonatal health.

**Fig. 3.**
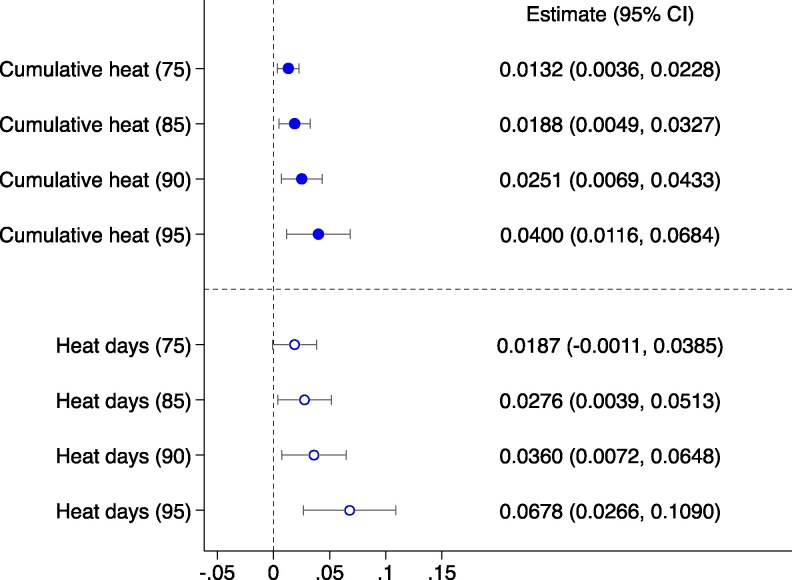
The impact of in utero extreme heat exposure on neonatal mortality. This figure displays the estimated impacts of prenatal extreme heat exposure on neonatal mortality among pregnant women, measured by *Cumulative heat (75, 85, 90, or 95)* (intensive level) and *Heat days (75, 85, 90, or 95)* (extensive level) using birth-level data. We controlled for DHS cluster, birth year, and 2° grid cell-birth month fixed effects. Control variables also include the monthly average precipitation of the DHS cluster, infant gender, birth order, and the age of the mother (including age squared) at the time of birth, as well as the mother's education level. SEs are clustered at the DHS cluster level in all specifications. Each bar represents a separate regression, and the error bars indicate 95% CIs.

To investigate the effects of prenatal exposure to high temperatures on neonatal mortality, we used neonate-level data and fitted a model that regressed neonatal mortality—measured as a binary variable indicating whether a neonate died before reaching 1 month of age. For ease of interpretation, we multiplied the coefficient by 1,000 to represent neonatal mortality per 1,000 live births, based on cumulative wet-bulb extreme heat exposure during the 9-month pregnancy period. The model controls for birth year and month, region, DHS cluster fixed effects, and mother and infant characteristics. The model specification is provided in expression (2) in the Materials and methods section, and the outcomes are shown in Fig. 3 and columns (1)–(3) of Table S6. These results offer a detailed view from an intensive margin perspective, quantifying total heat exposure intensity. A clear pattern emerged: as the threshold for daily abnormal heat exposure rises, from the 75th to the 95th percentile of seasonal temperatures over the previous 3 years, the impact on neonatal mortality intensifies. Specifically, the neonatal mortality rate increases from 0.0146 to 0.0436 as the temperature percentile threshold rises. This suggests that newborn health is increasingly compromised by higher prenatal exposure to extreme temperatures, with the severity of the impact rising alongside the extremity of the heat.

The analysis of the extensive margin reveals the impact of the duration of prenatal heat exposure on neonatal mortality. Unlike the intensive margin, which focuses on the overall intensity of extreme heat exposure measured by temperature, the extensive margin is based on the total number of days exposed to extreme heat. It emphasizes the length of exposure rather than its severity. Echoing the pattern identified through the intensive margin analysis, the extensive margin highlights a different aspect of exposure risk: as the criteria for defining abnormal extreme heat during pregnancy become more stringent, the negative effects on neonatal mortality intensify. Specifically, the data show that an additional 50 days of extreme heat exposure during the 9-month gestation period is associated with an increase of 1 to 4 neonatal deaths per 1,000 births within the first month of life. The consistency in exposure risk measured across both intensive and extensive margin analyses underscores the urgent need for comprehensive strategies that address not only the intensity of heat exposure but also its duration. Our analysis identifies a compounded extreme heat risk factor contributing to increased neonatal mortality.

We then studied whether there exist differences regarding neonatal health responses to prenatal extreme heat between rural and urban areas. Considering the systematic difference between rural and urban areas in terms of economic development, access to diverse income sources, and service infrastructure, which safeguard them from the detrimental effects of extreme heat so that urban populations posse more opportunities to mitigate climate risks induced by extreme heat (24), together with the significant neonatal mortality difference in our sample (rural area: 29.92 versus urban area: 27.40), we tested the heat impacts for rural and urban areas separately.

The results shown in Fig. S3 suggest that neonatal mortality in urban areas does not respond significantly to prenatal heat exposure, whereas rural areas show a pronounced effect. Specifically, as illustrated in Fig. S3a, the marginal effect of cumulative extreme heat increases neonatal mortality from 0.0184 to 0.0517 as the threshold for defining extreme heat rises. This corresponds to a 0.06–0.17% increase relative to the rural sample average of 29.92 deaths per 1,000 live births. Furthermore, an additional 50 days of exposure to extreme heat during pregnancy leads to 1.7–4.4 more neonatal deaths per 1,000 live births—an increase of 5.68–14.7% compared with the rural average (see columns (4)–(9) in Table S6 for details). Given that neonatal mortality in urban regions appears insensitive to prenatal heat exposure, we focus the remainder of our analysis on rural areas.

To test the reliability of our findings, we conducted supplementary analyses to validate the robustness of our models against systematic bias and data anomalies in the rural sample. For instance, in Eswatini (formerly known as Swaziland), we observed an unusually high neonatal mortality rate of 100% in rural areas, based on only 30 DHS observations (see Table S5 and Fig. S2). This rate is significantly higher than those observed in other countries. To account for this outlier, we repeated our analysis excluding Eswatini. The results, presented in Fig. S4 and Table S7, confirm that the exclusion of this outlier does not alter the overall pattern of our findings.

As part of our robustness tests, we adjusted our fixed-effects model by shifting from 2° grid cell by birth month fixed effects to country by birth month and 1° grid cell by birth month fixed effects. This refinement enhances our control for temporal and spatial variations by considering broader regional scopes, such as country or 1° grid cell by birth month. The results, presented in Fig. S5 and Table S8, confirm that the pattern and significance of our findings remain consistent despite these adjustments.

**Fig. 4.**
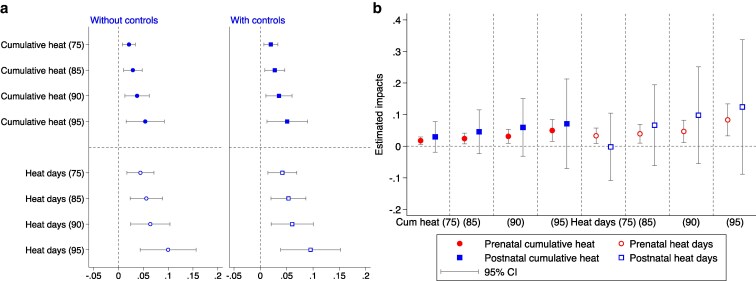
Robustness of heat exposure effects on neonatal mortality: accounting for migration and postnatal exposure. a) Regression coefficients from analyses restricted to mothers who remained in their current residence throughout the entire gestational period. Each bar represents a separate regression, where neonatal mortality—defined as 1,000 if an infant dies within the first month of life, and 0 otherwise—is regressed on *Cumulative heat* and *Heat days*, measured at the 75th, 85th, 90th, and 95th percentile thresholds. b) Regression coefficients for models that control for both prenatal and postnatal heat exposure, including heat exposure during the birth month. Each group of bars represents a separate regression, where neonatal mortality is regressed on prenatal *Cumulative heat* and *Heat days* at the same percentile thresholds, along with corresponding postnatal heat exposure variables. All regressions control for maternal and infant characteristics, including infant gender, birth order, mother's age (and age squared) at birth, and maternal education. Additional controls include monthly average precipitation in the DHS cluster during the 9-month pregnancy period, as well as DHS cluster and birth year fixed effects. SEs are clustered at the DHS cluster level. Error bars indicate 95% CIs.

To address potential concerns regarding accuracy of our exposure measures and the risk of endogeneity, we conducted a further sensitivity test relating to migration. If mothers had relocated during their pregnancy or were not present in the DHS clusters where they were initially residing, our exposure measure may be fully or partially compromised for such individuals. By cross-referencing DHS interview dates, birth dates, and duration of residence, we narrow our analysis to include only those mothers who spent their entire pregnancy within the same DHS cluster. The analysis, detailed in Fig. 4a here and Table S9, upholds our initial findings, confirming the detrimental impact of prenatal exposure to extreme heat on neonatal mortality.

As a further sensitivity test, we adjusted for postbirth heat exposure as an alternative method to eliminate the confounding effects associated with it. The outcomes, detailed in Fig. 4b and Table S10, show that after controlling for postbirth heat exposure, prenatal heat exposure remained significantly positive. Additionally, the coefficients for postbirth heat exposure variables were insignificant across all models, suggesting that heat exposure during the first month postbirth does not significantly impact neonatal mortality. The association between the extreme heat effects of climate change and infant mortality seems from our analysis to be confined to the in uterus rather than immediate neonatal stage.

We also tested the association of prenatal exposure to extreme cold and neonatal mortality, given that some research has identified extreme cold temperatures as a risk factor for mortality (25–27). Utilizing the same methodology as for extreme heat exposure, we developed indicators for cumulative cold exposure and the number of days experiencing extreme cold, based on the 5th, 10th, 15th, and 25th percentiles. A day’s temperature falling below these cold thresholds was classified as experiencing abnormal cold. Our analysis revealed that none of the eight extreme cold exposure metrics significantly affected neonatal mortality, confirming that it is extreme heat, rather than cold in Africa, that contributes to an increase in neonatal mortality rates (as shown in Fig. S6 and Table S11).

Our final sensitivity analysis modified the criteria for defining extreme high temperatures, extending the reference period from 3 to 5 years. The adjustment narrowed the scope of analysis to births from October 2008 to June 2020 and had the effect of defining extreme heat less conservatively, since a 3-year reference period is by definition less smoothed than a 5-year period. We found that the outcomes closely mirrored those obtained using the 3-year benchmark (highlighted in Fig. S7 and Table S12). We also show in Tables S13 and S14 that our results are also robust to the use of 10- and 18-year period.

### Mechanisms

Our analysis clearly indicates that prenatal exposure to elevated temperatures adversely affects neonatal health, impacting unborn children through their expectant mothers during the fetal period. To better understand the underlying mechanisms of these heat-induced consequences during pregnancy, we conducted two further analyses. First, we examined the relationship between extreme heat and the frequency of prenatal checkups, testing the hypothesis that extreme heat may discourage expectant mothers from attending clinic visits. Second, we conducted a subsample analysis to investigate the association between in utero heat exposure and neonatal survival rates based on the type of drinking water and sanitation facilities, testing the hypothesis that access to these basic utilities can improve neonatal health by reducing exposure to pathogens.

**Fig. 5.**
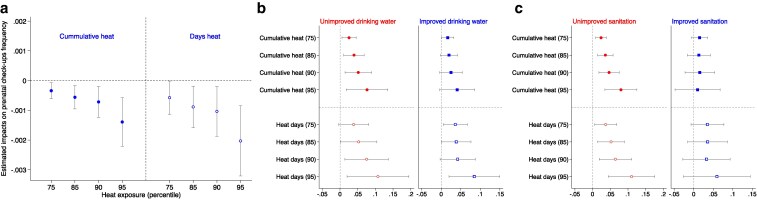
Mechanisms linking in utero extreme heat exposure to neonatal mortality. a) The “prenatal checkups prevention” channel. The figure displays the estimated impacts of prenatal extreme heat exposure on rural pregnant women, measured by *Cumulative heat (75, 85, 90, or 95)* and *Heat days (75, 85, 90, or 95)*, on the frequency of hospital visits for medical care during pregnancy. b and c) The results of the “disease channel” test, plotting the subsample analysis coefficients based on the household's drinking water source and sanitation facility type. Control variables include the monthly average precipitation of the DHS cluster, birth gender (excluded in (a) as the infant's gender is unknown during pregnancy), birth order, the mother's age (and age squared) at childbirth, the mother's education, DHS cluster, birth year, and 2° grid cell-birth month fixed effects. SEs are clustered at the DHS cluster level in all specifications. Each bar represents a separate regression, with error bars indicating 95% CIs.

Prenatal checkups are crucial for monitoring fetal health and obtaining expert medical advice on fetal development (28). However, high ambient temperatures may discourage pregnant women from attending regular prenatal checkups to avoid heat exposure, potentially compromising fetal well-being (29, 30). We utilized model (3) from the Materials and methods section to investigate this phenomenon. Our analysis, depicted in Fig. 5a and Table S15, shows that an increase in cumulative heat exposure intensity—measured by both cumulative heat (75 to 95) and heat days (75–95)—correlates with a decline in the frequency of prenatal checkups, supporting the “prenatal checkups prevention” hypothesis.

Additionally, sustainable access to safe water and sanitation significantly benefits maternal and infant health by reducing the prevalence of diarrheal and other diseases (31, 32). Hot and humid conditions promote the growth of harmful bacteria and parasites (33, 34), particularly in tropical and arid regions of Africa, where essential facilities are often lacking, and sanitation conditions inadequate (35, 36). The use of unimproved water sources, such as unprotected wells or springs, increases the risk of pathogen infection. Similarly, inadequate sanitation facilities can heighten exposure to bacteria and pathogens, underscoring the importance of access to safe drinking water and proper sanitation for maternal health (28, 37, 38).

Subsample analyses based on water and sanitation factors, as detailed in Fig. 5b and c (with further details in Table S16), reveal that households using unimproved water sources or sanitation facilities experience significantly lower neonatal survival rates. Specifically, the lack of access to improved drinking water or sanitation facilities is associated with increased neonatal mortality risks, which intensify with higher heat exposure. A clear pattern emerges, showing that as temperature thresholds rise, the risks to neonatal survival increase. In conclusion, our data are consistent with elevated temperatures not only deterring pregnant women from accessing medical care but also increasing the risk of infection from harmful bacteria, as a result of a compounding effect of poor sanitation and water in extreme heat locations.

### Heterogeneous impact across subpopulations

To better understand the effects of prenatal extreme heat exposure, particularly in rural regions, we explored its heterogeneous impacts across different populations. This analysis identifies the groups most vulnerable to extreme heat, offering valuable insights for targeted adaptation strategies and policy interventions. We examined subpopulations based on household poverty (wealth), electricity availability, and the mother's education level and modeled these effects. The results are presented in Fig. 5 and Table S15.

The adverse effects of prenatal extreme heat on neonatal mortality were most significant among populations in the poor or middle wealth classes, with effects ranging from 0.0186 to 0.0549, exceeding average impacts for the whole rural sample (39). Wealthier households, however, showed an insignificant variation from the mean, possibly due to their ability to employ effective adaptation strategies and access to quality healthcare, nutrition, and living conditions (40–44) (Fig. 5a, columns (1) and (2) in Table S15).

Lack of electricity, a crucial factor in mitigating heat discomfort through air conditioners and electric fans, was also examined as an independent factor (45). As expected, populations without electricity access experienced significantly higher neonatal mortality rates in response to heat exposure, highlighting the importance of electricity for adapting to rising temperatures (Fig. S8b, columns (3) and (4) in Table S17).

Maternal education was assessed as a “soft” contextual factor along with infrastructure factors, since knowledge and traditional values can affect use of infrastructure. Children born to mothers with lower education levels were more at risk of neonatal mortality by high temperatures than to mothers with secondary or higher education. This suggests that education provides critical health-related knowledge and awareness necessary for protecting the fetus from harm as the impact of climate change in heat-health risk areas progresses. The mitigating effect of knowledge on the adverse impacts of extreme heat (Fig. S8c, columns (5) and (6) in Table S17) seems to us to be an important leverage point for public health policy.

## Materials and methods

### Data

#### Daily dry bulb temperature

Our data source on land surface temperature (LST) is the Global Seamless 1 km Resolution Daily Land Surface Temperature Dataset (GSDLST) maintained by Iowa State University (13). Combining Moderate Resolution Imaging Spectroradiometer (MODIS) satellite images and a state-of-art spatiotemporal gap-filling framework, the dataset has global coverage on near-surface temperature at a 1-km resolution for the 2003–2022 period on a daily basis. Compared with other MODIS-based products on LST, GSDLST's high spatiotemporal resolution enables us to precisely map heat exposure to neonatal mortality, thus allowing us to make credible causal inference on the impact of abnormally hot temperature exposure on neonatal mortality in Africa. GSDLST also fixes cloud contamination, a common cause of missing values in other LST datasets.

#### Daily relative daily relative humidity

In evaluating the relationship between a mother's exposure to abnormal heat during pregnancy and a newborn's death within 1 month, we employed wet-bulb temperature, which concurrently considers temperature and relative humidity (22, 46). This is essential because relative humidity impedes the human body's ability to sweat, a crucial mechanism for cooling itself and alleviating heat stress. As demonstrated by several recent studies, human perception of thermal comfort is more accurately captured by wet-bulb temperature, as it accounts for the interaction effect between ambient temperature and relative humidity on the human body (21–23, 46). To this end, we obtained daily relative humidity data from the agrometeorological dataset curated by the European Centre for Medium-Range Weather Forecasts through the Copernicus Climate Change Service. This dataset provides information on relative humidity at a 10-km resolution with daily, global coverage. We combined ambient temperature data from the GSDLST with the relative humidity information to derive the wet-bulb temperature.

#### Monthly precipitation

Our data on precipitation comes from AfroGrid, a dataset that offers environmental information at the 0.5 × 0.5 decimal degrees grid cell by month level for the period of 1989–2020 (47).

#### Neonatal mortality

We obtained data on neonatal mortality in Africa from the DHS. Conducted in developing countries since the 1980s, the DHS are nationally representative, repeated cross-sectional surveys that assess reproductive and health behaviors. Administered by local implementing agencies, the DHS select a random set of participating households and gathers a wide range of health-related information at both the household and individual levels. Moreover, the survey asks each interviewed woman to provide their complete birth histories, including the birth date (i.e. month and year) for each child ever born and the age of death in months if the child did not survive. Based on this information, we defined *neonatal mortality* as a dummy variable equal to 1 when a child was reported to have died within the first month following birth (age at death is 0 months), and 0 otherwise. We then multiplied this value by 1,000 to facilitate interpretation. The average neonatal mortality rate in our sample is 29 deaths per 1,000 births.

Furthermore, for each child ever born, we extracted additional child characteristics (birth year, birth month, gender, birth order), maternal characteristics (education, birth year, frequency of prenatal checkups), and household characteristics (source of drinking water, type of sanitation, type of residence, electricity availability, wealth quintile) from the DHS. These were utilized for constructing control variables and heterogeneous factors.

Since the DHS also provide information on survey locations (i.e. clusters), we constructed a panel dataset of birth year and month by location for the 2003–2020 period. We incorporated all surveys conducted in Africa and restricted our focus to those children for whom we had comprehensive temperature data for the 9 months preceding their births (i.e. those born after October 2006). Furthermore, we excluded infants who were alive when interviewed and younger than 1 month. Our final sample included 32,694 survey clusters in 3,500 2° grid cells across 33 countries, covering ∼883,623 births during the period from October 2006 to June 2020. The summary statistics are presented in Table S2. And Tables S3 and S4 show summary statistics for rural and urban sample, respectively.

#### Extreme temperature exposure measures construction

We first calculated daily wet-bulb temperature using daily dry bulb temperature and relative humidity from 2003 to 2020 following the Stull calculation:


(1) $$\begin{array}{rl} wt & =t\times \arctan\;\left[0.151977\times {\left(rh+8.313658\right)}^{\frac{1}{2}}\right]\\ & \;+\arctan\;(t+rh)-\arctan\;(rh-1.676331)\\ & \;+0.00391838\times rh^{\frac{3}{2}}\times \arctan(0.023101\times rh)-4.686035 \end{array}$$

where *t* represents the dry bulb temperature in degree Celsius and *rh* is the relative humidity in percentage format, ranging from 0 to 100. *wt* (wet-bulb temperature) calculated using the above formula is also in degree Celsius.

For every season *s* (*s* = 1, 2, 3, 4: season 1 includes January, February, and March; season 2 includes April, May, and June; season 3 includes July, August, and September; season 4 includes October, November, and December) in year *t* (*t* = 2006 to 2020), we first obtained the 5th, 10th, 15th, 25th, 75th, 85th, 90th, and 95th percentile (*P5*, *P10*, *P25*, *P75*, *P85*, *P90*, and *P95*, respectively) of wet-bulb temperatures over the past 3 years (i.e. *t*−3 to *t*−1). For daily temperatures *wt*, values exceeding the 75th percentile (P75) of the corresponding year-season are classified as extreme heat (P75), with similar classifications for the 85th (P85), 90th (P90), and 95th (P95) percentiles. Conversely, if *wt* falls below the 25th percentile (P25), it is defined as extreme cold (P25), with analogous definitions for the 15th (P15), 10th (P10), and 5th (P5) percentiles.

For a child born in month *m*, we calculate the in utero cumulative exposure to extreme heat or cold by summarizing the part of daily temperature that exceeds the specified percentiles over the previous 9 months (from *m*−9 to *m*−1). The following shows an example, if daily temperature exceeds the 75th percentile (P75).


$$\mathrm{Extreme}\;\;\mathrm{heat}\;(75{)}_{m}=\;\overset{m-9}{\underset{m-1}{\sum }}\;(\mathrm{Daily}\;\mathrm{temperature}-75\mathrm{th}\;\;\mathrm{percentile}\;\mathrm{temperature})$$

This results in cumulative heat or cold exposure, classified by the specified percentiles (75, 85, 90, and 95 for heat and 25, 15, 10, and 5 for cold).

Additionally, we quantify the total number of days with an extreme heat or cold temperature, referred to as *extreme heat* or *cold days exposure* (heat or cold days for the respective percentiles). The following shows an example, if daily temperature exceeds the 75th percentile (P75).


$$\mathrm{Extreme}\;\;\mathrm{heat}\;\;\mathrm{days}\;(75{)}_{m}=\;\overset{m-9}{\underset{m-1}{\sum }}\;(\mathrm{Number}\;\mathrm{of}\;\mathrm{days}\;\;\mathrm{with}\;\;\mathrm{temperature}\;\;\mathrm{above}\;75\;\;\mathrm{percentile})$$

The cumulative exposure reflects the intensive margin, while the count of exposure days represents the extensive margin.

### Empirical strategy

#### Baseline regression

We first explored the impact of mother's extreme heat exposure during the 9-month pregnancy period on neonatal mortality using the following model.


(2) $$\begin{array}{rl} \mathrm{Neonatal}\;\;\mathrm{mortalit}y_{icrmt} & =\alpha_{0}+\alpha_{1}\mathrm{Extreme}\;\;\mathrm{heat}\;\;\mathrm{exposur}e_{icrmt}\\ & \;+\beta \mathrm{Control}s_{icrmt}+\mathrm{DHS}\;\;\mathrm{cluste}r_{c}\\ & \;+\mathrm{Birth}\;\;\mathrm{yea}r_{t}+\mathrm{Region}\\ & \;-\mathrm{birth}\;\;\mathrm{mont}h_{rm}+\varepsilon_{icrmt} \end{array}$$

Here, *i* indexes child, *c* indexes DHS cluster, and *r* denotes the 2° grid cell, which corresponds to an administrative area similar to an average county. The indices *m* and *t* refer to the child's birth month and year, respectively. The dependent variable is *Neonatal mortality*, which equals 1 when a child was reported to have died within the first month after birth, and 0 otherwise. This variable is then multiplied by 1,000 for the ease of interpretation. The primary explanatory variable is $\mathrm{Extreme}\;\;\mathrm{heat}\;\;\mathrm{exposur}e_{icrmt}$, which represents the infant's in utero exposure to extreme heat. For the intensive margin, this includes *Cumulative heat (75) (or 85, 90, 95)*, while for the extensive margin, it encompasses *Heat days (75) (or 85, 90, 95).* The measure of interest is $\alpha_{1}$, which indicates the estimated impact of in utero extreme heat exposure on neonatal mortality.

We included in model (2) weather-related, children- and mother-level control variables ($\mathrm{Control}s_{icrmt}$) that may confound the results. Additionally, we included DHS cluster-level monthly average precipitation during the 9-month pregnancy period. Children characteristics include children's gender and birth order, while mother characteristics include mother's age when she gave birth, age squared, and her education level. Moreover, we added DHS cluster fixed effects ($\mathrm{DHS}\;\mathrm{cluste}r_{c}$) to absorb DHS cluster-level unobserved time-invariant factors such as geographic characteristics and culture, birth year fixed effects ($\mathrm{Birth}\;\;\mathrm{yea}r_{t}$) to control for time trends, and the 2° grid cell-birth month fixed effects ($\mathrm{Region}-\mathrm{birth}\;\mathrm{mont}h_{rm}$) to control for the average of region time-variant variation in the main results. We also controlled for country-birth month or 1° grid cell-birth month fixed effects in model (2) in our additional robustness tests. We clustered the SEs at the DHS cluster level allowing for arbitrary correlations between any two error terms within the same DHS cluster. $\varepsilon_{icrmt}$ is the residual.

#### Prenatal checkups prevention channel test

Next, we explored the mechanisms underlying the negative effect of in utero extreme heat exposure on neonatal mortality. To investigate whether extreme heat exposure prevents mothers from visiting hospitals for medical checkups before childbirth, leading to an increase in neonatal mortality rates, we regressed the following model.


(3) $$\begin{array}{rl} \mathrm{No}.\;\;\mathrm{of}\;\;\mathrm{prenatal}\;\mathrm{check}s_{icrmt} & =\alpha_{0}+\alpha_{1}\mathrm{Extreme}\;\;\mathrm{heat}\;\mathrm{exposur}e_{icrmt}\\ & \;+\beta \mathrm{Control}s_{icrmt}+\mathrm{DHS}\;\mathrm{cluste}r_{c}\\ & \;+\mathrm{Birth}\;\;\mathrm{yea}r_{t}+\mathrm{Region}\\ & \;-\mathrm{birth}\;\;\mathrm{mont}h_{rm}+\varepsilon_{icrmt} \end{array}$$

where the dependent variable is the number of visits that pregnant women make to the hospital or health clinics for medical care during their pregnancy. The control variables $\mathrm{Control}s_{icrmt}$ include DHS cluster-level monthly average precipitation, birth order, mother's age at childbirth, age squared, and her education level. The other variables are the same as that in model (2). If $\alpha_{1}$ is significantly negative, it would support the proposed channel.

#### Heterogeneous effects

We further employed model (2) to document the heterogeneity of the effects of extreme heat exposure through subsample analyses based on household and mother characteristics.

Subpopulation analyses based on the source of drinking water and the type of sanitation facility allow us to investigate the disease channel. In accordance with WHO standards, we defined the following as *improved water* sources: piped water, tube wells or boreholes, protected wells or springs, rainwater, tanker trucks, carts with small tanks, and bottled water. All other sources were classified as *unimproved water*. *Improved sanitation* facilities include nonshared facilities that flush or pour flush to a piped sewer system, septic tanks, or pit latrines; ventilated improved pit latrines; pit latrines with a slab; and composting toilets. In contrast, facilities that flush to a known location but not to a sewer system, septic tank, or pit latrine (i.e. those that flush to “somewhere else”), pit latrines without slabs or open pits, bucket toilets, hanging toilets or latrines, and other similar facilities were classified as *unimproved sanitation*.

We also examined the heterogeneous effects by considering the type of residence (rural versus urban), household wealth quintile (comparing the “poorest, poorer, or middle” categories to the “richer or richest” categories), electricity availability, and the mother's education level (distinguishing between “no education or primary” and “secondary or higher”).

## Discussion

Our study delves into the consequences of prenatal extreme heat exposure, incorporating relative humidity, on neonatal mortality in Africa from 2006 to 2020. Rural areas suffer significantly, while urban regions are not sensitive to extreme heat. In terms of rural area, we discovered that extreme heat increases neonatal mortality not only through encouraging the growth of bacteria and pathogens but also via restricting pregnant women's access to essential prenatal medical checkups, leading to a significant increase in neonatal mortality rates. These findings enrich the existing literature and address critical policy questions.

Our findings contribute to a growing body of research examining environmental determinants of early-life mortality in SSA. For example, Heft-Neal et al. (48) document the adverse effects of poor air quality on infant mortality across the region, while Pullabhotla et al. (49) show that short-term exposure to biomass fire pollution also increases infant mortality. These studies underscore the vulnerability of infants to environmental hazards and highlight the urgent need for targeted policy responses. Our study complements this literature by focusing on a different but increasingly important climate-related risk: extreme heat. In doing so, we shift the emphasis to in utero exposure and neonatal mortality, rather than broader infant mortality outcomes. This focus allows for a more precise attribution of the mortality effects to prenatal conditions and identifies specific pathways—such as access to sanitation and professional health care—that may mitigate these risks. Together, this emerging evidence base points to a set of compounding environmental threats that demand urgent and localized intervention in Africa's ongoing effort to meet child survival goals.

Our study is also closely related to the literature examining the impact of heat exposure on child mortality, particularly the works of Banerjee and Maharaj (28) and Geruso and Spears (38), which focus on infant and neonatal mortality. Banerjee and Maharaj (28) explore the effects of ambient temperature on neonatal mortality in India and find a positive association between high-temperature exposure during both pregnancy and the first month after birth and neonatal deaths. However, their analysis does not account for humidity, a crucial factor that affects the body's ability to regulate temperature through sweating. Moreover, because they combine prenatal and postnatal exposures, their study cannot clearly isolate the effect of in utero heat exposure—an important distinction for designing targeted interventions during pregnancy versus after birth.

Geruso and Spears (38) address some of these limitations by using wet-bulb temperature, which incorporates humidity, to study heat exposure during the first month of life across 53 developing countries. However, their use of low-resolution temperature data (27.75 km grids) matched to village-level mortality data may introduce measurement error and thus produce imprecise estimates. They also apply a uniform definition of “extreme heat” across all countries, failing to account for local adaptation to heat. Furthermore, while their findings are significant, they focus solely on postnatal exposure and do not offer specific policy recommendations.

Our study builds on and extends this literature in several important ways. By leveraging high-resolution (1 km) temperature data, we accurately align heat exposure with DHS village clusters and use fixed effects to control for unobserved heterogeneity. We define extreme heat using village-specific wet-bulb temperature thresholds, accounting for local adaptation. Focusing on in utero exposure, we show it has a significant impact on neonatal mortality independent of postnatal exposure. Additionally, we investigate whether access to professional prenatal health care can mitigate this risk, and find strong evidence that it does—offering practical, low-cost policy insights for improving maternal and infant health in SSA under climate stress.

While previous studies have highlighted the health impacts of high temperatures, such as increased hospitalization (50), morbidity (51), and mortality rates (43, 52–54), as well as the alteration of infectious disease transmission patterns (51, 55, 56), our work zeroes in on maternal health's heightened sensitivity to temperature changes. By filling a notable gap in the literature outlined above (48, 49, 57–69), our study complements existing research on other infant health outcomes and aligns with findings that link prenatal air pollution exposure to adverse health effects in infants and neonates.

Our findings offer actionable insights for improving neonatal health and survival in the face of high ambient temperatures, especially for rural regions that lacking infrastructure and mitigation approaches. We suggest that health organizations could mitigate these effects by promoting awareness of prenatal care, offering home checkup services, or increasing the number of health centers. Additionally, local governments play a crucial role in ensuring the availability of clean drinking water and improved sanitation facilities, as our analysis indicates that households lacking these amenities are particularly vulnerable to extreme heat.

Our heterogeneity analysis sheds light on the subpopulations most at risk from extreme prenatal heat exposure, including those of lower wealth, without access to electricity, and mothers with limited education. Addressing these disparities requires both short-term measures, such as providing guidance and financial support for vulnerable households, and long-term strategies, like enhancing women's education and investing in infrastructure. These efforts are essential for mitigating the impacts of global warming on vulnerable populations (37, 70).

While our heterogeneity analysis highlights meaningful variation in the relationship between prenatal heat exposure and neonatal mortality across subpopulations—such as by access to improved water, sanitation, electricity, and maternal education—we acknowledge that these characteristics are often correlated with broader dimensions of household wealth and infrastructure. As such, we cannot fully disentangle whether the observed differential effects stem from these specific factors themselves or from other unobserved socioeconomic conditions that covary with them. Therefore, we interpret these results as suggestive and indicative of relative vulnerability, rather than conclusive evidence of causal moderation by any single characteristic. Nonetheless, these patterns are valuable for identifying groups at elevated risk and can inform the targeting of adaptation strategies.

## Conclusion

While our study offers valuable insights into the indirect mechanisms linking prenatal heat exposure to neonatal mortality in rural areas, further research is needed to deepen our understanding of these relationships and to explore the short-term effects of heat exposure on human health. This future research will be crucial for developing a comprehensive approach to safeguarding well-being in the context of climate change, especially those more vulnerable.

## Supplementary Material

pgaf240_Supplementary_Data

## Data Availability

Data and code can be found at https://osf.io/xntkz/ and doi:10.17605/OSF.IO/XNTKZ.

## Notes

a For more detail, see https://www.dhsprogram.com/Who-We-Are/About-Us.cfm.
