# Supplementary material for: In utero exposure to extreme heat increases neonatal mortality
Source: PNAS Nexus. 2025 Aug 19;4(8):pgaf240. doi: 10.1093/pnasnexus/pgaf240 (PMC12362353; doi:10.1093/pnasnexus/pgaf240)
Supplement: pgaf240_Supplementary_Data [file pgaf240_supplementary_data.pdf]

Supplementary Materials for
In Utero Exposure to Extreme Heat Increases Neonatal Mortality

Tenghui Wang[1,#], Jiafu An[2,#,*], Bin Chen[2], Chris John Webster[2], Peng Gong[3], Chen Lin[1*]

[1] Faculty of Business and Economics, The University of Hong Kong, Hong Kong SAR, China.

[2] Faculty of Architecture, The University of Hong Kong, Hong Kong SAR, China.

[3] Department of Geography and Department of Earth Sciences, The University of Hong Kong, Hong Kong SAR, China.

#These authors contributed equally to this work

*Corresponding author. Jiafu An (jiafuan@hku.hk); Chen Lin (chenlin1@hku.hk)

Figure S1. Study regions and annual distribution of live births

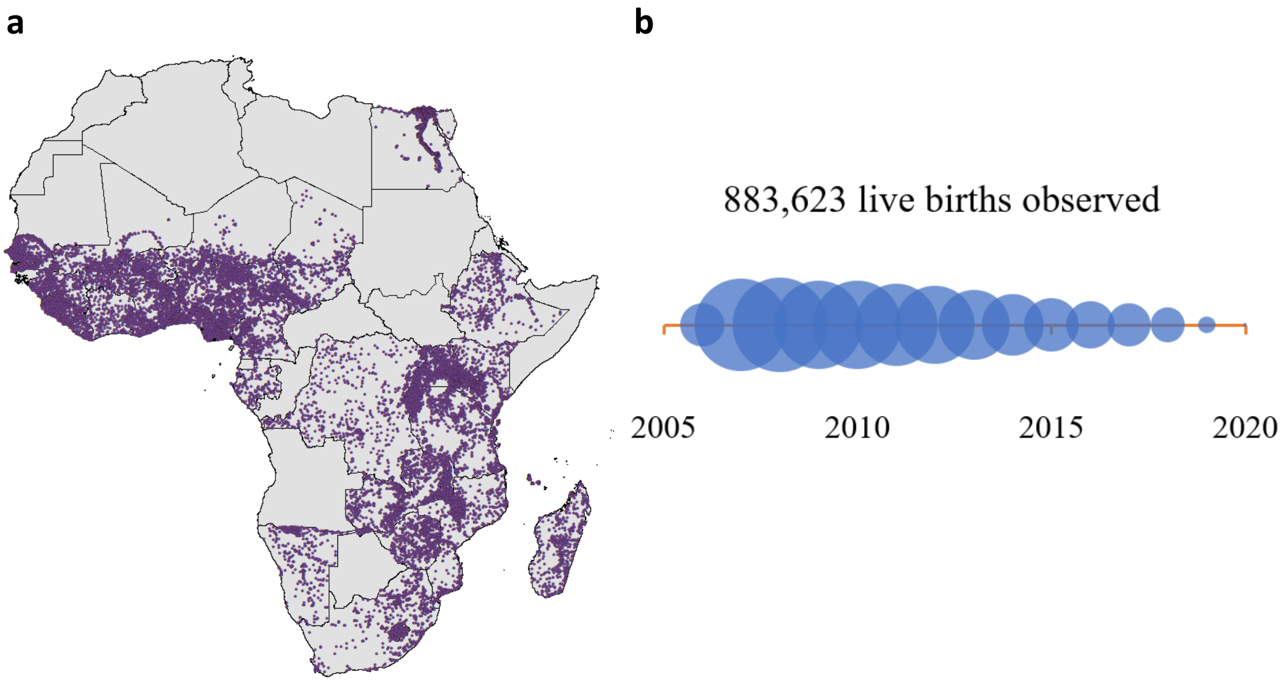

Note: This figure provides an overview of the study regions in Africa and the birth data used in our analysis. (a) The locations of DHS clusters included in the analysis sample. (b) The number of live births recorded each year in the sample, spanning from October 2006 to June 2020. The lower number of observations in 2006 is due to the sample only covering three months of births for that year.

Figure S2. Neonatal mortality per 1,000 births by country

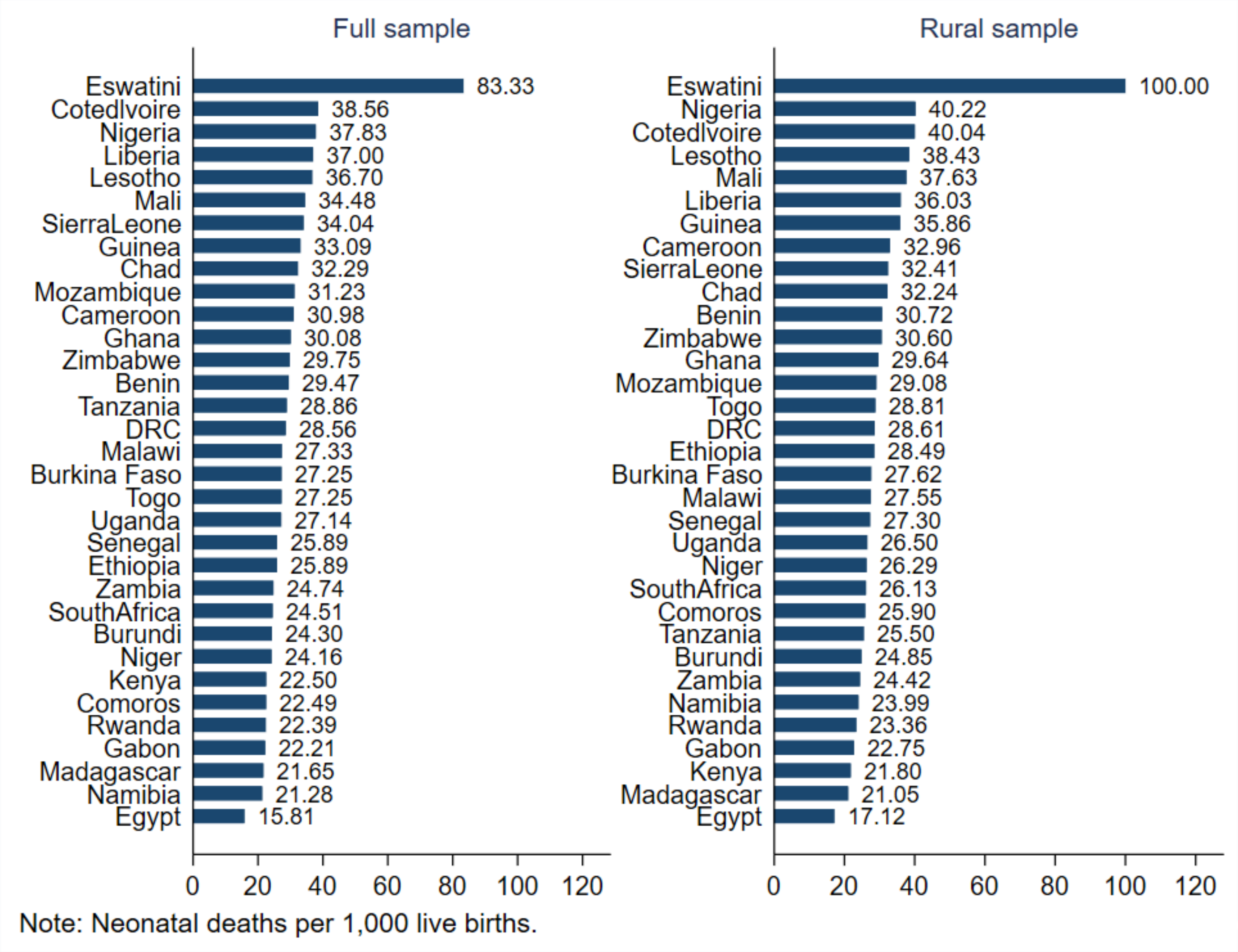

Note: This figure presents neonatal mortality rates per 1,000 live births by country for both the full sample and the rural sample in Africa. The data reflects the complete sample used in our study.

Fig. S3. The impact of in utero extreme heat exposure on neonatal mortality, by rural-urban region

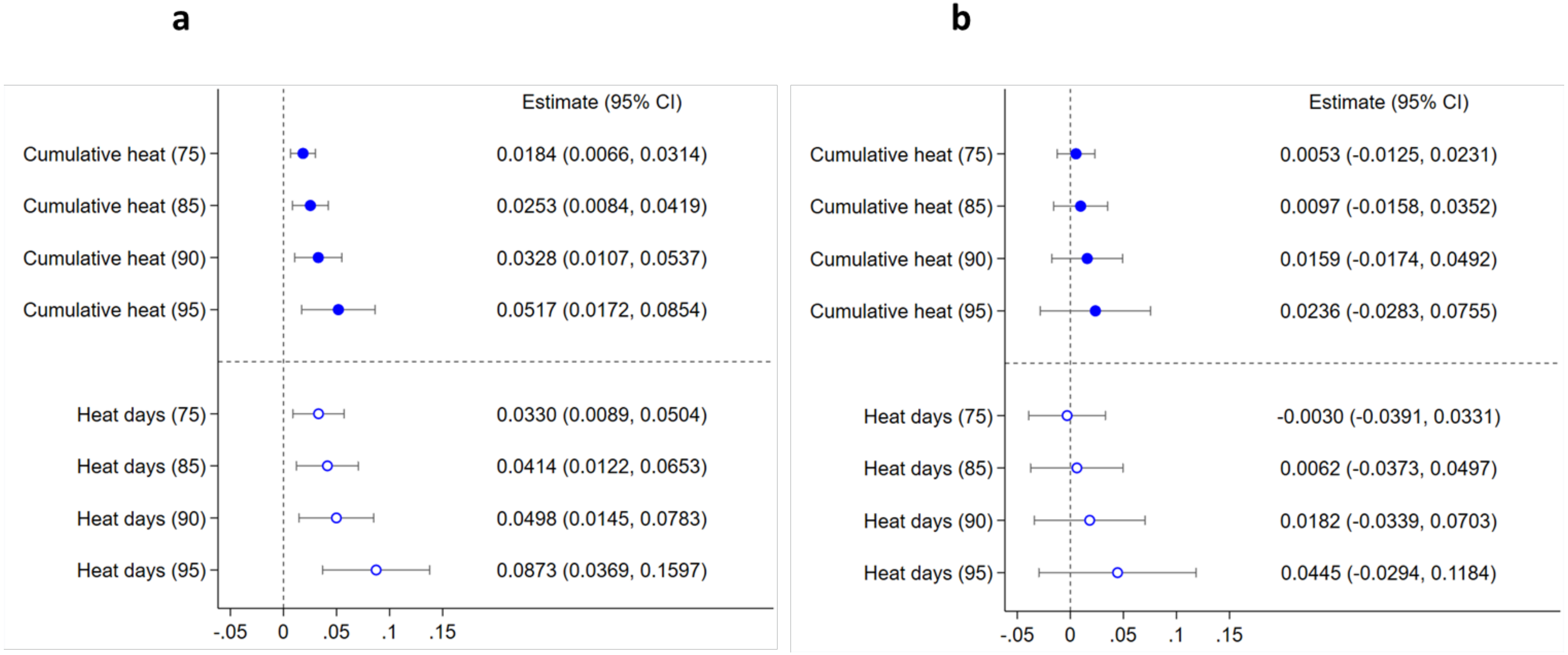

Note: This figure displays the estimated impacts of prenatal extreme heat exposure on neonatal mortality among pregnant women, measured by *Cumulative heat (75, 85, 90, or 95)* (intensive level) and *Heat days (75, 85, 90, or 95)* (extensive level) for rural sample (Panel a) and urban sample (Panel b) separately, using birth-level data. We controlled for DHS cluster, birth year, and 2-degree grid cell-birth month fixed effects. Control variables also include the monthly average precipitation of the DHS cluster, infant gender, birth order, and the age of the mother (including age squared) at the time of birth, as well as the mother's education level. Standard errors are clustered at the DHS cluster level in all specifications. Each bar represents a separate regression, and the error bars indicate 95% confidence intervals.

Figure S4. Regression coefficients of neonatal mortality, excluding Eswatini

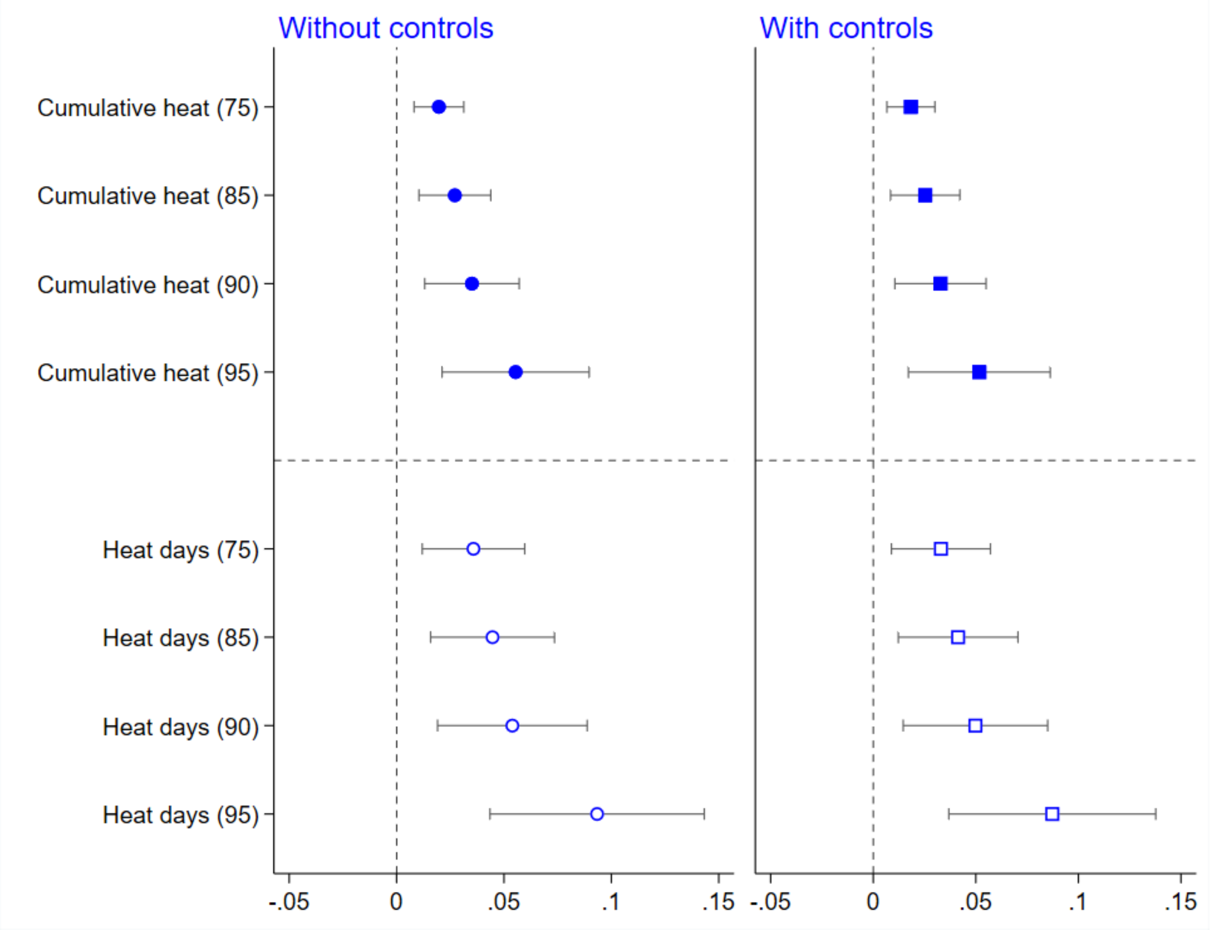

Note: This figure depicts the regression coefficients after excluding observations from Eswatini. Detailed regression results are presented in Extended Data Table 7. Each bar represents a separate regression where we regressed neonatal mortality—a variable equal to 1,000 if an infant dies before reaching 1 month of age, and 0 otherwise—on explanatory variables including *Cumulative heat* at thresholds of 75, 85, 90, and 95 degrees, and *Heat days* at the same thresholds. The regressions control for DHS cluster, birth year, and 2-degree grid cell-birth month fixed effects. Additional controls include monthly average precipitation in the DHS cluster, infant gender, birth order, mother's age (and age squared) at birth, and maternal education. Standard errors are clustered at the DHS cluster level in all models, and the error bars represent 95% confidence intervals.

Figure S5. Regression coefficients of neonatal mortality with alternative fixed effects

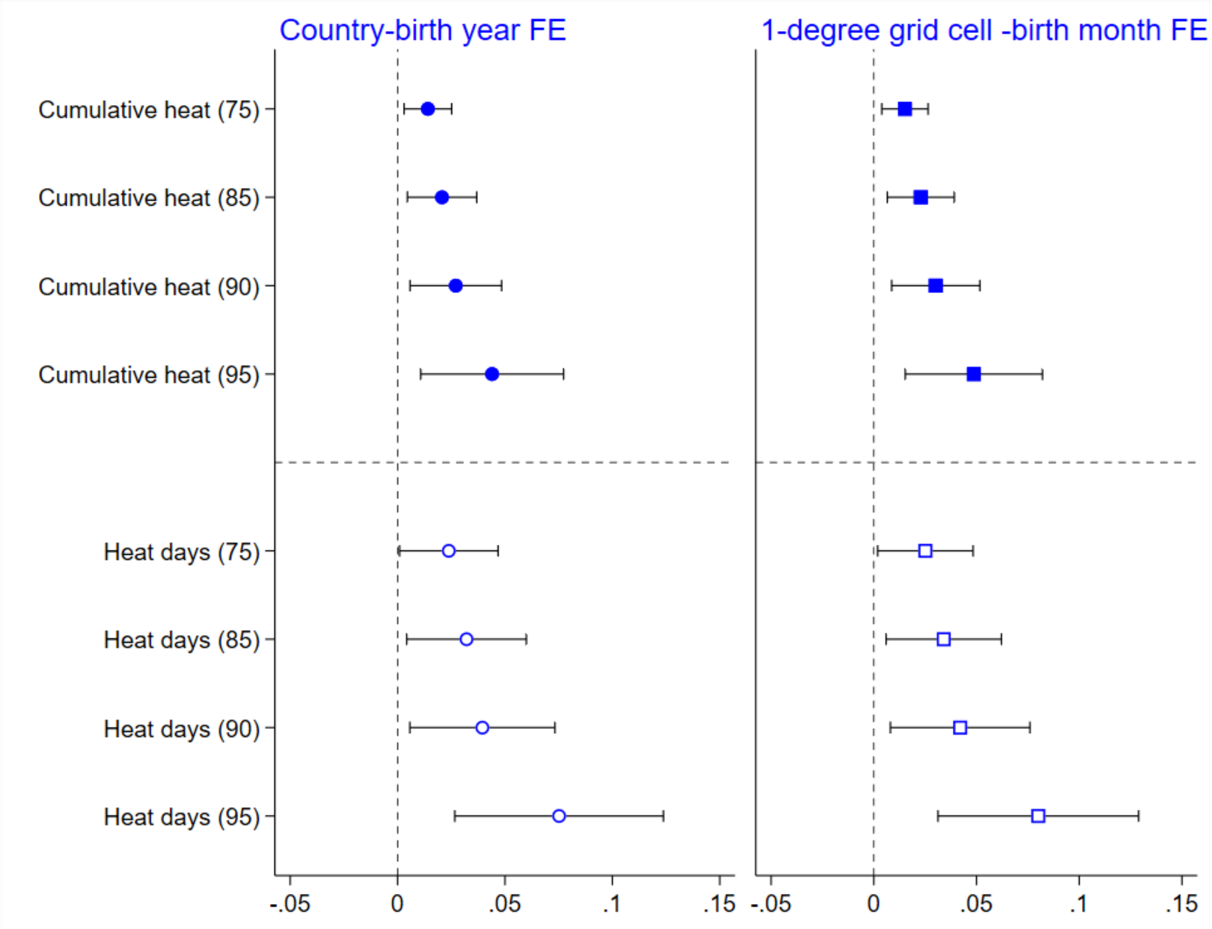

Note: This figure presents the regression coefficients using alternative fixed effects, as shown in Extended Data Table 8. Each bar represents a separate regression where we regressed neonatal mortality—a variable equal to 1,000 if an infant dies before reaching 1 month of age, and 0 otherwise—on explanatory variables including *Cumulative heat* at thresholds of 75, 85, 90, and 95 degrees, as well as *Heat days* at the same thresholds. In all specifications, we controlled for monthly average precipitation in the DHS cluster during the 9-month pregnancy period, infant gender, birth order, mother's age (and age squared) at birth, maternal education, DHS cluster, and birth year fixed effects. Standard errors are clustered at the DHS cluster level. Additionally, we controlled for either country-birth month or 1-degree grid cell-birth month fixed effects separately. The error bars represent 95% confidence intervals.

Figure S6. Regression coefficients of prenatal extreme cold exposure on neonatal mortality

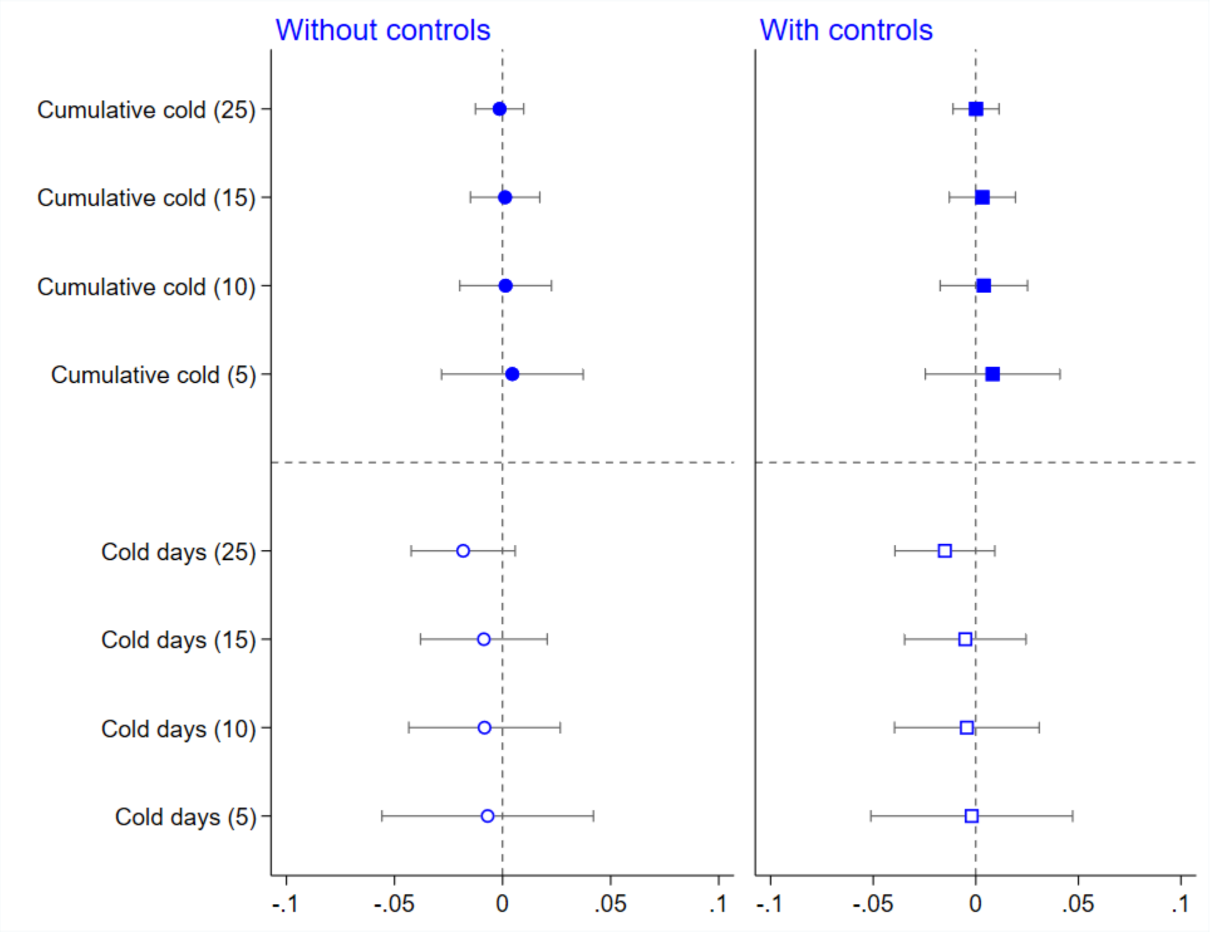

Note: This figure presents the regression coefficients exploring the impact of prenatal extreme cold exposure on neonatal mortality as part of a robustness test. Detailed regression results are provided in Extended Data Table 11. Each bar represents a separate regression where we regressed neonatal mortality—a variable equal to 1,000 if an infant dies before reaching 1 month of age, and 0 otherwise—on explanatory variables including *Cumulative cold* at thresholds of 75, 85, 90, and 95 degrees, and *Cold days* at the same thresholds. The regressions control for DHS cluster, birth year, and 2-degree grid cell-birth month fixed effects. Additional controls include monthly average precipitation in the DHS cluster, infant gender, birth order, mother's age (and age squared) at birth, and maternal education. Standard errors are clustered at the DHS cluster level in all specifications, and the error bars represent 95% confidence intervals.

Figure S7. Regression coefficients of neonatal mortality using a 5-year benchmark for extreme heat exposure

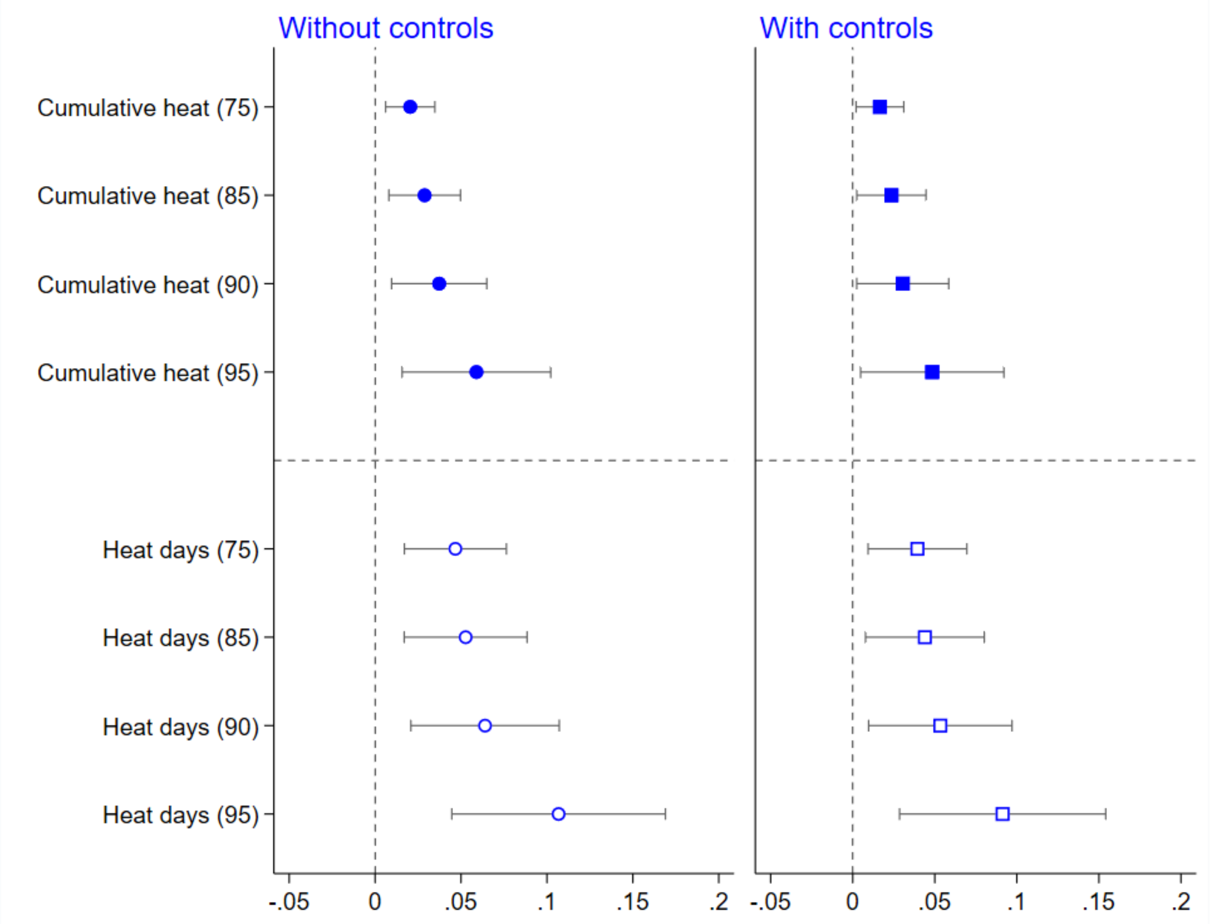

Note: This figure presents the regression coefficients after modifying the benchmark temperature to the past 5 years (instead of 3) when calculating extreme heat exposure as part of a robustness test. Detailed regression results are provided in Extended Data Table 12. Each bar represents a separate regression where we regressed neonatal mortality—a variable equal to 1,000 if an infant dies before reaching 1 month of age, and 0 otherwise—on explanatory variables including *Cumulative heat* at thresholds of 75, 85, 90, and 95 degrees, as well as *Heat days* at the same thresholds. The regressions control for DHS cluster, birth year, and 2-degree grid cell-birth month fixed effects. Additional controls include monthly average precipitation in the DHS cluster, infant gender, birth order, mother's age (and age squared) at birth, and maternal education. Standard errors are clustered at the DHS cluster level, and the error bars represent 95% confidence intervals.

Fig. S8. Heterogeneous impacts by subpopulations

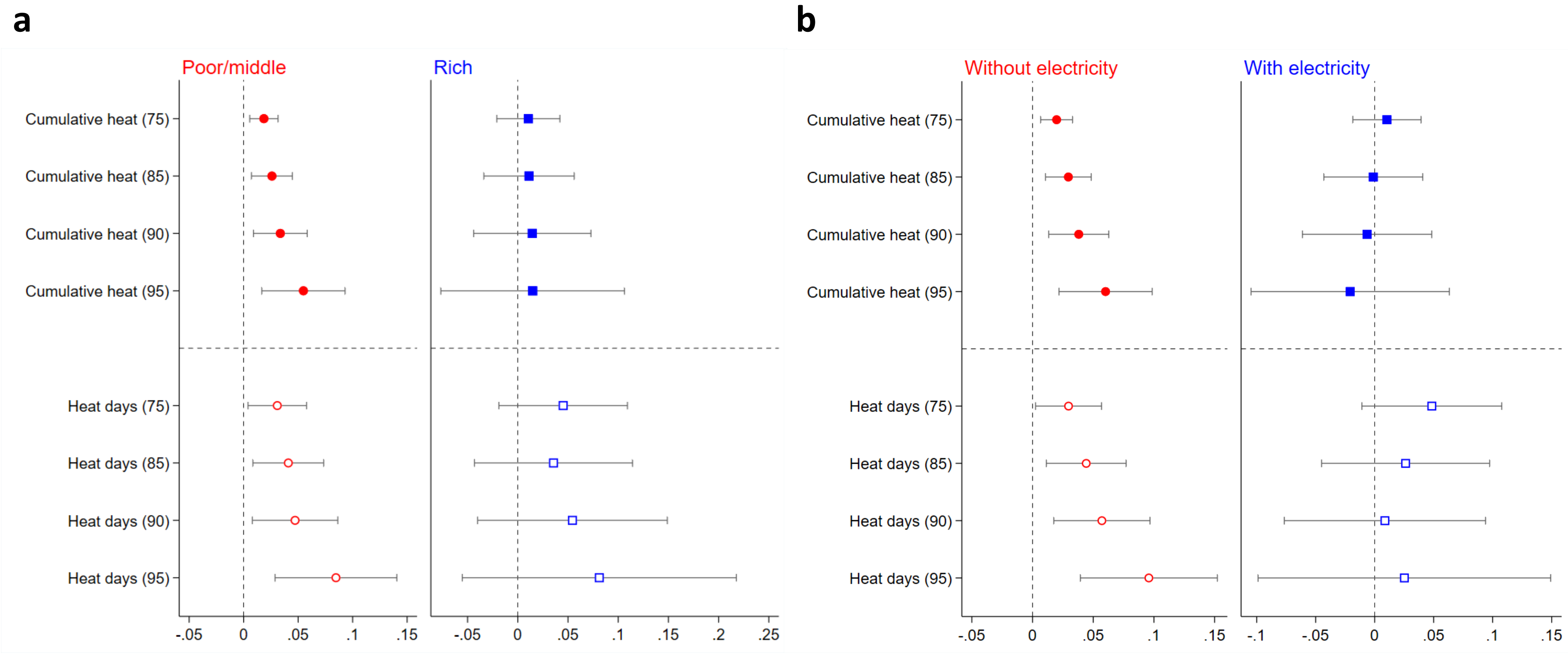

c

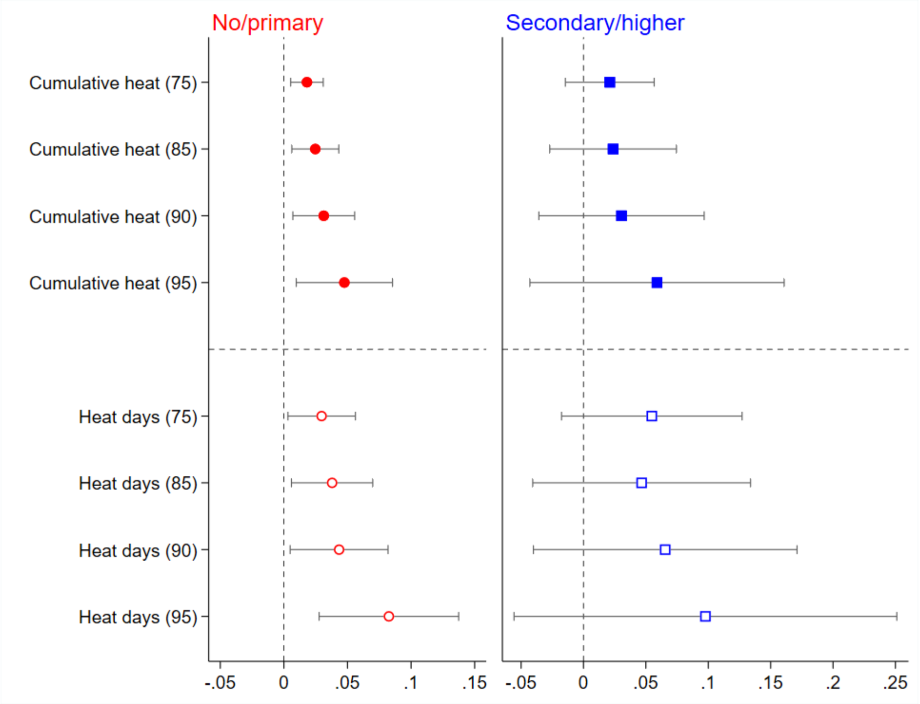

Note: This figure presents a subsample analysis based on rural sample focusing on characteristics of the household or mother, encompassing (a) household wealth level, (b) availability of electricity, and (c) education level of the mother. Each bar represents the results from a separate regression. Error bars indicate 95% confidence intervals.

Table S1. Variable definition

Variable	Definition
Neonatal mortality	It equals to 1000 if an infant dies before reaching the age of 1 month, and 0 otherwise.
Cumulative heat (75)	The summation of daily abnormal heat (the part where daily wet bulb temperature is higher than the 75th percentile of that belonging to the same season over the past three years) exposure over the total 9-month pregnancy period.
Cumulative heat (85)	The summation of daily abnormal heat (the part where daily wet bulb temperature is higher than the 85th percentile of that belonging to the same season over the past three years) exposure over the total 9-month pregnancy period.
Cumulative heat (90)	The summation of daily abnormal heat (the part where daily wet bulb temperature is higher than the 90th percentile of that belonging to the same season over the past three years) exposure over the total 9-month pregnancy period.
Cumulative heat (95)	The summation of daily abnormal heat (the part where daily wet bulb temperature is higher than the 95th percentile of that belonging to the same season over the past three years) exposure over the total 9-month pregnancy period.
Heat days (75)	The total days when daily wet bulb temperature is higher than the 75th percentile of that belonging to the same season over the past three years during the total 9-month pregnancy period.
Heat days (85)	The total days when daily wet bulb temperature is higher than the 85th percentile of that belonging to the same season over the past three years during the total 9-month pregnancy period.
Heat days (90)	The total days when daily wet bulb temperature is higher than the 90th percentile of that belonging to the same season over the past three years during the total 9-month pregnancy period.
Heat days (95)	The total days when daily wet bulb temperature is higher than the 95th percentile of that belonging to the same season over the past three years during the total 9-month pregnancy period.
Cumulative heat post (75)	The summation of daily abnormal heat (the part where daily wet bulb temperature is higher than the 75th percentile of that belonging to the same season over the past three years) exposure at the birth month.
Cumulative heat post (85)	The summation of daily abnormal heat (the part where daily wet bulb temperature is higher than the 85th percentile of that belonging to the same season over the past three years) exposure at the birth month.
Cumulative heat post (90)	The summation of daily abnormal heat (the part where daily wet bulb temperature is higher than the 90th percentile of that belonging to the same season over the past three years) exposure at the birth month.
Cumulative heat post (95)	The summation of daily abnormal heat (the part where daily wet bulb temperature is higher than the 95th percentile of that belonging to the same season over the past three years) exposure at the birth month.
Heat days post (75)	The total days when daily wet bulb temperature is higher than the 75th percentile of that belonging to the same season over the past three years at the birth month.
Heat days post (85)	The total days when daily wet bulb temperature is higher than the 85th percentile of that belonging to the same season over the past three years at the birth month.

Heat days post (90)	The total days when daily wet bulb temperature is higher than the 90th percentile of that belonging to the same season over the past three years at the birth month.
Heat days post (95)	The total days when daily wet bulb temperature is higher than the 95th percentile of that belonging to the same season over the past three years at the birth month.
Cumulative cold (25)	The summation of daily abnormal heat (the part where daily wet bulb temperature is lower than the 25th percentile of that belonging to the same season over the past three years) exposure over the total 9-month pregnancy period.
Cumulative cold (15)	The summation of daily abnormal heat (the part where daily wet bulb temperature is lower than the 15th percentile of that belonging to the same season over the past three years) exposure over the total 9-month pregnancy period.
Cumulative cold (10)	The summation of daily abnormal heat (the part where daily wet bulb temperature is lower than the 10th percentile of that belonging to the same season over the past three years) exposure over the total 9-month pregnancy period.
Cumulative cold (5)	The summation of daily abnormal heat (the part where daily wet bulb temperature is lower than the 5th percentile of that belonging to the same season over the past three years) exposure over the total 9-month pregnancy period.
Cold days (25)	The total days when daily wet bulb temperature is lower than the 25th percentile of that belonging to the same season over the past three years during the total 9-month pregnancy period.
Cold days (15)	The total days when daily wet bulb temperature is lower than the 15th percentile of that belonging to the same season over the past three years during the total 9-month pregnancy period.
Cold days (10)	The total days when daily wet bulb temperature is lower than the 10th percentile of that belonging to the same season over the past three years during the total 9-month pregnancy period.
Cold days (5)	The total days when daily wet bulb temperature is lower than the 5th percentile of that belonging to the same season over the past three years during the total 9-month pregnancy period.
Average precipitation	Monthly average precipitation over the past 9 months during pregnancy.
Female	A dummy variable equals to 1 if the infant is female, and 0 otherwise.
Birth order	The order certain infant was born for certain mother.
Mother age	The mother's age when she gave birth.
No education	A dummy variable equals to 1 if the mother did not receive education, and 0 otherwise.
Primary	A dummy variable equals to 1 if the mother holds primary level education, and 0 otherwise.
Secondary	A dummy variable equals to 1 if the mother holds secondary level education, and 0 otherwise.
Higher	A dummy variable equals to 1 if the mother holds higher level education, and 0 otherwise.
No. of prenatal checks	The number of visits the mother go to the hospital for medical care during pregnancy.

Table S2. Summary statistics: Full sample

Variable	Obs.	Mean	Median	Sd	P5	P10	P25	P75	P90	P95	Min	Max
Neonatal mortality	883623	29.1448	0.0000	168.2124	0.0000	0.0000	0.0000	0.0000	0.0000	0.0000	0.0000	1000.0000
Cumulative heat (75)	883623	91.6763	81.8651	51.7516	27.1325	36.5901	55.1545	117.5835	161.2021	191.1729	0.0000	933.1162
Cumulative heat (85)	883623	48.7687	41.0364	33.5298	10.5181	15.3987	25.6330	63.0891	92.0470	114.9544	0.0000	538.2551
Cumulative heat (90)	883623	30.9951	24.5550	24.7822	4.9292	7.8630	14.2259	40.2201	62.1838	80.2898	0.0000	338.2718
Cumulative heat (95)	883623	15.1735	10.5038	15.4638	1.0847	2.2759	5.1750	19.5936	33.5584	45.8919	0.0000	206.0087
Heat days (75)	883623	66.6979	65.0000	24.1033	32.0000	39.0000	51.0000	81.0000	98.0000	109.0000	0.0000	202.0000
Heat days (85)	883623	40.6214	38.0000	18.6841	15.0000	19.0000	28.0000	51.0000	65.0000	75.0000	0.0000	158.0000
Heat days (90)	883623	27.6891	25.0000	15.1087	8.0000	11.0000	17.0000	36.0000	48.0000	56.0000	0.0000	132.0000
Heat days (95)	883623	14.5275	12.0000	10.3726	2.0000	4.0000	7.0000	19.0000	28.0000	35.0000	0.0000	101.0000
Cumulative heat post (75)	883623	10.5596	5.2414	14.9812	0.0000	0.0000	1.0960	13.6380	27.8311	40.0456	0.0000	224.7459
Cumulative heat post (85)	883623	5.6652	2.1957	9.2460	0.0000	0.0000	0.0000	7.0654	15.7122	23.4686	0.0000	153.5474
Cumulative heat post (90)	883623	3.6283	1.0418	6.6683	0.0000	0.0000	0.0000	4.2817	10.2987	16.0569	0.0000	125.2941
Cumulative heat post (95)	883623	1.7946	0.0451	4.0726	0.0000	0.0000	0.0000	1.8001	5.2021	8.7970	0.0000	91.7061
Heat days post (75)	883623	7.5390	6.0000	6.8646	0.0000	0.0000	2.0000	12.0000	18.0000	21.0000	0.0000	31.0000
Heat days post (85)	883623	4.6282	3.0000	5.1709	0.0000	0.0000	0.0000	7.0000	12.0000	15.0000	0.0000	31.0000
Heat days post (90)	883623	3.1788	2.0000	4.0845	0.0000	0.0000	0.0000	5.0000	9.0000	12.0000	0.0000	31.0000
Heat days post (95)	883623	1.6873	1.0000	2.7112	0.0000	0.0000	0.0000	2.0000	5.0000	7.0000	0.0000	29.0000
Cumulative cold (25)	883623	101.4897	90.6120	59.1109	27.6161	38.3368	59.8421	130.8640	179.5443	214.5204	0.0000	751.1954
Cumulative cold (15)	883623	54.3845	45.8852	38.0442	10.4086	15.7325	27.5449	71.9546	103.3307	126.4528	0.0000	476.9953
Cumulative cold (10)	883623	34.3244	27.5124	27.2437	4.7566	7.8554	15.3602	45.8132	69.2800	85.7529	0.0000	367.1910
Cumulative cold (5)	883623	16.6273	11.7070	16.6176	0.9992	2.1942	5.4941	22.0617	36.8938	49.7330	0.0000	329.4588
Cold days (25)	883623	71.5441	70.0000	25.4270	33.0000	41.0000	55.0000	88.0000	104.0000	114.0000	0.0000	211.0000
Cold days (15)	883623	44.4065	42.0000	20.1778	15.0000	21.0000	30.0000	57.0000	71.0000	80.0000	0.0000	170.0000
Cold days (10)	883623	30.7137	29.0000	16.3911	8.0000	12.0000	19.0000	40.0000	53.0000	61.0000	0.0000	167.0000
Cold days (5)	883623	16.4975	14.0000	11.2206	2.0000	4.0000	8.0000	22.0000	32.0000	38.0000	0.0000	158.0000
Average precipitation	883623	97.2570	90.8667	58.5067	14.8444	29.9333	57.8333	124.8444	166.5778	212.9667	0.0000	379.8222
Female	883623	0.4930	0.0000	0.5000	0.0000	0.0000	0.0000	1.0000	1.0000	1.0000	0.0000	1.0000
Birth order	883623	3.5894	3.0000	2.3634	1.0000	1.0000	2.0000	5.0000	7.0000	8.0000	1.0000	18.0000
Mother age	883623	30.7005	30.0000	7.1971	20.0000	22.0000	25.0000	36.0000	41.0000	44.0000	15.0000	50.0000
No education	883623	0.4505	0.0000	0.4975	0.0000	0.0000	0.0000	1.0000	1.0000	1.0000	0.0000	1.0000
Primary	883623	0.3202	0.0000	0.4666	0.0000	0.0000	0.0000	1.0000	1.0000	1.0000	0.0000	1.0000

Secondary	883623	0.1989	0.0000	0.3991	0.0000	0.0000	0.0000	0.0000	1.0000	1.0000	0.0000	1.0000
Higher	883623	0.0305	0.0000	0.1719	0.0000	0.0000	0.0000	0.0000	0.0000	0.0000	0.0000	1.0000
No. of prenatal checks	393334	4.1532	4.0000	3.3175	0.0000	0.0000	3.0000	5.0000	7.0000	10.0000	0.0000	90.0000

Note: This table presents summary statistics of the births from October 2006 to July 2020. Variable definitions are shown in Table 1.

Table S3. Summary statistics: Rural sample

Variable	Obs.	Mean	Median	Sd	P5	P10	P25	P75	P90	P95	Min	Max
Neonatal mortality	627278	29.8958	0.0000	170.3001	0.0000	0.0000	0.0000	0.0000	0.0000	0.0000	0.0000	1000.0000
Cumulative heat (75)	627278	92.6739	82.7107	51.1177	29.2561	38.3342	56.4511	118.2716	161.7225	191.6146	0.0000	512.7108
Cumulative heat (85)	627278	49.1697	41.4222	33.1984	11.3787	16.1585	26.2314	63.3095	92.0382	114.8718	0.0000	375.1604
Cumulative heat (90)	627278	31.1726	24.7536	24.5847	5.3727	8.2520	14.5879	40.2577	62.0522	80.0983	0.0000	281.7758
Cumulative heat (95)	627278	15.2040	10.5566	15.3932	1.2152	2.4090	5.3109	19.5360	33.3679	45.6784	0.0000	204.9540
Heat days (75)	627278	66.8544	65.0000	23.8323	33.0000	39.0000	51.0000	81.0000	98.0000	109.0000	0.0000	202.0000
Heat days (85)	627278	40.6945	38.0000	18.5284	15.0000	20.0000	28.0000	51.0000	65.0000	75.0000	0.0000	158.0000
Heat days (90)	627278	27.7072	25.0000	14.9965	8.0000	11.0000	17.0000	36.0000	48.0000	56.0000	0.0000	132.0000
Heat days (95)	627278	14.4879	12.0000	10.2960	3.0000	4.0000	7.0000	19.0000	28.0000	35.0000	0.0000	101.0000
Cumulative heat post (75)	627278	10.6651	5.3385	15.0275	0.0000	0.0000	1.1244	13.8364	28.1171	40.2294	0.0000	207.7027
Cumulative heat post (85)	627278	5.7171	2.2469	9.2657	0.0000	0.0000	0.0042	7.1646	15.8384	23.5433	0.0000	152.4296
Cumulative heat post (90)	627278	3.6542	1.0682	6.6718	0.0000	0.0000	0.0000	4.3389	10.3781	16.1009	0.0000	125.2941
Cumulative heat post (95)	627278	1.8015	0.0530	4.0611	0.0000	0.0000	0.0000	1.8260	5.2304	8.7874	0.0000	90.6646
Heat days post (75)	627278	7.5585	6.0000	6.8799	0.0000	0.0000	2.0000	12.0000	18.0000	22.0000	0.0000	31.0000
Heat days post (85)	627278	4.6416	3.0000	5.1822	0.0000	0.0000	1.0000	7.0000	12.0000	15.0000	0.0000	31.0000
Heat days post (90)	627278	3.1852	2.0000	4.0876	0.0000	0.0000	0.0000	5.0000	9.0000	12.0000	0.0000	31.0000
Heat days post (95)	627278	1.6865	1.0000	2.7058	0.0000	0.0000	0.0000	2.0000	5.0000	7.0000	0.0000	29.0000
Cumulative cold (25)	627278	101.6476	91.0234	58.2791	28.5058	38.9732	60.4578	130.7920	178.5474	213.0757	0.0000	751.1954
Cumulative cold (15)	627278	54.3195	46.0088	37.5179	10.7644	15.9890	27.7692	71.8083	102.4919	125.0968	0.0000	476.9953
Cumulative cold (10)	627278	34.2838	27.5472	26.9719	4.9712	7.9902	15.5031	45.7475	68.6846	85.0805	0.0000	338.4671
Cumulative cold (5)	627278	16.6042	11.7155	16.5350	1.0851	2.2600	5.5399	21.9718	36.6411	49.6618	0.0000	175.7508
Cold days (25)	627278	71.9262	71.0000	25.2418	34.0000	41.0000	55.0000	88.0000	105.0000	115.0000	0.0000	203.0000
Cold days (15)	627278	44.6677	43.0000	20.1512	16.0000	21.0000	30.0000	57.0000	72.0000	81.0000	0.0000	168.0000
Cold days (10)	627278	30.9144	29.0000	16.4189	8.0000	12.0000	19.0000	40.0000	53.0000	61.0000	0.0000	145.0000
Cold days (5)	627278	16.6165	14.0000	11.2823	3.0000	4.0000	8.0000	22.0000	32.0000	38.0000	0.0000	109.0000
Average precipitation	627278	95.6128	90.0778	56.2146	16.8778	31.0778	57.6778	122.1000	161.3667	204.4444	0.0000	379.8222
Female	627278	0.4932	0.0000	0.5000	0.0000	0.0000	0.0000	1.0000	1.0000	1.0000	0.0000	1.0000
Birth order	627278	3.7850	3.0000	2.4291	1.0000	1.0000	2.0000	5.0000	7.0000	8.0000	1.0000	18.0000
Mother age	627278	30.7181	30.0000	7.3001	20.0000	21.0000	25.0000	36.0000	41.0000	44.0000	15.0000	50.0000

No education	627278	0.5144	1.0000	0.4998	0.0000	0.0000	0.0000	1.0000	1.0000	1.0000	0.0000	1.0000
Primary	627278	0.3380	0.0000	0.4730	0.0000	0.0000	0.0000	1.0000	1.0000	1.0000	0.0000	1.0000
Secondary	627278	0.1361	0.0000	0.3429	0.0000	0.0000	0.0000	0.0000	1.0000	1.0000	0.0000	1.0000
Higher	627278	0.0115	0.0000	0.1065	0.0000	0.0000	0.0000	0.0000	0.0000	0.0000	0.0000	1.0000
No. of prenatal checks	274691	3.7089	3.0000	3.0025	0.0000	0.0000	2.0000	5.0000	6.0000	8.0000	0.0000	90.0000

Note: This table presents summary statistics of the births in rural areas from October 2006 to July 2020. Variable definitions are shown in Table 1.

Table S4. Summary statistics: Urban sample

Variable	Obs.	Mean	Median	Sd	P5	P10	P25	P75	P90	P95	Min	Max
Neonatal mortality	256345	27.3070	0.0000	162.9766	0.0000	0.0000	0.0000	0.0000	0.0000	0.0000	0.0000	1000.0000
Cumulative heat (75)	256345	89.2352	79.6934	53.1923	22.7508	32.3199	51.8178	115.9134	159.8991	190.1696	0.0000	933.1162
Cumulative heat (85)	256345	47.7874	40.0291	34.3076	8.6137	13.5621	24.0249	62.5115	92.0683	115.0986	0.0000	538.2551
Cumulative heat (90)	256345	30.5609	24.0278	25.2536	3.8620	6.9204	13.3396	40.1213	62.5217	80.7541	0.0000	338.2718
Cumulative heat (95)	256345	15.0989	10.3610	15.6350	0.7888	1.9681	4.8273	19.7269	33.9961	46.3950	0.0000	206.0087
Heat days (75)	256345	66.3151	65.0000	24.7498	30.0000	38.0000	50.0000	81.0000	98.0000	109.0000	0.0000	194.0000
Heat days (85)	256345	40.4425	38.0000	19.0586	13.0000	19.0000	28.0000	51.0000	65.0000	75.0000	0.0000	151.0000
Heat days (90)	256345	27.6447	25.0000	15.3798	7.0000	11.0000	17.0000	36.0000	48.0000	56.0000	0.0000	120.0000
Heat days (95)	256345	14.6245	12.0000	10.5573	2.0000	4.0000	7.0000	19.0000	29.0000	35.0000	0.0000	92.0000
Cumulative heat post (75)	256345	10.3015	5.0064	14.8642	0.0000	0.0000	1.0284	13.1764	27.1474	39.6005	0.0000	224.7459
Cumulative heat post (85)	256345	5.5381	2.0753	9.1965	0.0000	0.0000	0.0000	6.8294	15.4402	23.3121	0.0000	153.5474
Cumulative heat post (90)	256345	3.5652	0.9804	6.6594	0.0000	0.0000	0.0000	4.1495	10.1126	15.9623	0.0000	123.3079
Cumulative heat post (95)	256345	1.7779	0.0270	4.1008	0.0000	0.0000	0.0000	1.7383	5.1230	8.8208	0.0000	91.7061
Heat days post (75)	256345	7.4913	6.0000	6.8269	0.0000	0.0000	2.0000	12.0000	18.0000	21.0000	0.0000	31.0000
Heat days post (85)	256345	4.5956	3.0000	5.1429	0.0000	0.0000	0.0000	7.0000	12.0000	15.0000	0.0000	31.0000
Heat days post (90)	256345	3.1632	2.0000	4.0767	0.0000	0.0000	0.0000	5.0000	9.0000	12.0000	0.0000	31.0000
Heat days post (95)	256345	1.6893	1.0000	2.7244	0.0000	0.0000	0.0000	2.0000	5.0000	7.0000	0.0000	28.0000
Cumulative cold (25)	256345	101.1034	89.5795	61.0972	25.3510	36.7458	58.3114	131.0314	182.0612	218.0697	0.0000	566.3803
Cumulative cold (15)	256345	54.5436	45.5940	39.3020	9.4656	15.1090	26.9861	72.3172	105.7131	129.5758	0.0000	383.8953
Cumulative cold (10)	256345	34.4237	27.4357	27.8975	4.1938	7.5158	15.0004	45.9699	70.6328	87.2848	0.0000	367.1910
Cumulative cold (5)	256345	16.6838	11.6858	16.8180	0.8060	2.0256	5.3869	22.2848	37.4695	49.8567	0.0000	329.4588
Cold days (25)	256345	70.6091	69.0000	25.8507	32.0000	40.0000	54.0000	87.0000	104.0000	114.0000	0.0000	211.0000
Cold days (15)	256345	43.7674	42.0000	20.2286	14.0000	20.0000	30.0000	56.0000	71.0000	80.0000	0.0000	170.0000
Cold days (10)	256345	30.2227	28.0000	16.3127	8.0000	11.0000	19.0000	40.0000	52.0000	60.0000	0.0000	167.0000
Cold days (5)	256345	16.2061	14.0000	11.0628	2.0000	4.0000	8.0000	22.0000	31.0000	38.0000	0.0000	158.0000
Average precipitation	256345	101.2805	93.1333	63.5902	9.9111	26.8444	58.2000	131.8889	181.8667	230.0000	0.0000	379.8222
Female	256345	0.4926	0.0000	0.4999	0.0000	0.0000	0.0000	1.0000	1.0000	1.0000	0.0000	1.0000
Birth order	256345	3.1110	3.0000	2.1196	1.0000	1.0000	1.0000	4.0000	6.0000	7.0000	1.0000	18.0000
Mother age	256345	30.6575	30.0000	6.9386	20.0000	22.0000	25.0000	35.0000	40.0000	43.0000	15.0000	49.0000

No education	256345	0.2941	0.0000	0.4557	0.0000	0.0000	0.0000	1.0000	1.0000	1.0000	0.0000	1.0000
Primary	256345	0.2766	0.0000	0.4473	0.0000	0.0000	0.0000	1.0000	1.0000	1.0000	0.0000	1.0000
Secondary	256345	0.3523	0.0000	0.4777	0.0000	0.0000	0.0000	1.0000	1.0000	1.0000	0.0000	1.0000
Higher	256345	0.0770	0.0000	0.2665	0.0000	0.0000	0.0000	0.0000	0.0000	1.0000	0.0000	1.0000
No. of prenatal checks	118643	5.1820	4.0000	3.7550	1.0000	2.0000	3.0000	6.0000	9.0000	12.0000	0.0000	50.0000

Note: This table presents summary statistics of the births in urban areas from October 2006 to July 2020. Variable definitions are shown in Table 1.

Table S5. List of DHS surveys in the sample

Country	Survey year	Observations		
		Full sample	Rural	Urban
Benin	2011-2012, 2017-2018	40,579	25,097	15,482
Burkina Faso	2010	11,411	8,907	2,504
Burundi	2010, 2016-2017	31,891	26,721	5,170
Cameroon	2011, 2018	30,603	17,687	12,916
Chad	2014-2015	31,743	25,220	6,523
Comoros	2012	3,512	2,239	1,273
Cote d'lvoire	2011-2012	7,935	5,270	2,665
Democratic Republic of the Congo	2007, 2013-2014	25,074	17,265	7,809
Egypt	2008, 2014	25,748	15,365	10,383
Eswatini	2006-2007	60	30	30
Ethiopia	2016	3,515	2,773	742
Gabon	2012	6,349	2,418	3,931
Ghana	2008, 2014	10,305	6,175	4,130
Guinea	2012, 2018	25,473	18,433	7,040
Kenya	2008-2009, 2014	35,548	24,450	11,098
Lesotho	2009, 2014	7,002	5,412	1,590
Liberia	2007, 2019-2020	14,864	10,241	4,623
Madagascar	2008-2009	5,635	4,702	933
Malawi	2010, 2015-2016	46,508	40,111	6,397
Mali	2006, 2012, 2018	33,494	25,484	8,010
Mozambique	2011	10,791	7,358	3,433
Namibia	2006, 2013	6,907	3,794	3,113
Niger	2012	13,823	10,840	2,983
Nigeria	2008, 2013, 2018	127,374	85,554	41,820
Rwanda	2010, 2014-2015, 2019-2020	40,011	32,282	7,729
Senegal	2011, 2013, 2014, 2015, 2016, 2017, 2018, 2019	114,291	80,062	34,229
SierraLeone	2008, 2013, 2019	42,772	29,621	13,151
South Africa	2016	6,570	3,062	3,508
Tanzania	2010, 2015	21,693	16,979	4,714
Togo	2013-2014	10,056	7,324	2,732
Uganda	2011, 2016	35,963	29,322	6,641
Zambia	2007, 2013, 2018	41,669	27,636	14,033
Zimbabwe	2010-2011, 2015	14,454	9,444	5,010
Total		883,623	627,278	256,345

Note: This table represents the surveys and the number of observations across countries by type of residence that we used in the study.

Table S6. Impact of in utero extreme heat exposure on neonatal mortality

Dependent variable	Neonatal mortality								
	Full sample			Rural sample			Urban sample		
	(1)	(2)	(3)	(4)	(5)	(6)	(7)	(8)	(9)
Cumulative heat (75)	0.0146***	0.0149***	0.0132***	0.0197***	0.0199***	0.0184***	0.0069	0.0076	0.0053
	(0.0049)	(0.0049)	(0.0049)	(0.0059)	(0.0059)	(0.0060)	(0.0090)	(0.0090)	(0.0091)
Cumulative heat (85)	0.0206***	0.0211***	0.0188***	0.0271***	0.0274***	0.0253***	0.0116	0.0126	0.0097
	(0.0070)	(0.0070)	(0.0071)	(0.0085)	(0.0085)	(0.0086)	(0.0129)	(0.0129)	(0.0130)
Cumulative heat (90)	0.0274***	0.0280***	0.0251***	0.0351***	0.0353***	0.0328***	0.0183	0.0195	0.0159
	(0.0092)	(0.0092)	(0.0093)	(0.0112)	(0.0112)	(0.0113)	(0.0169)	(0.0169)	(0.0170)
Cumulative heat (95)	0.0436***	0.0442***	0.0400***	0.0554***	0.0555***	0.0517***	0.0271	0.0290	0.0236
	(0.0144)	(0.0144)	(0.0145)	(0.0175)	(0.0175)	(0.0176)	(0.0264)	(0.0263)	(0.0265)
Heat days (75)	0.0217**	0.0224**	0.0187*	0.0358***	0.0360***	0.0330***	0.0009	0.0022	-0.0030
	(0.0099)	(0.0099)	(0.0101)	(0.0122)	(0.0122)	(0.0123)	(0.0183)	(0.0183)	(0.0184)
Heat days (85)	0.0307**	0.0317***	0.0276**	0.0447***	0.0449***	0.0414***	0.0098	0.0118	0.0062
	(0.0120)	(0.0120)	(0.0121)	(0.0147)	(0.0147)	(0.0149)	(0.0220)	(0.0220)	(0.0222)
Heat days (90)	0.0397***	0.0408***	0.0360**	0.0539***	0.0540***	0.0498***	0.0220	0.0244	0.0182
	(0.0145)	(0.0145)	(0.0147)	(0.0178)	(0.0178)	(0.0180)	(0.0265)	(0.0264)	(0.0266)
Heat days (95)	0.0730***	0.0741***	0.0678***	0.0934***	0.0929***	0.0873***	0.0488	0.0524	0.0445
	(0.0208)	(0.0207)	(0.0210)	(0.0255)	(0.0254)	(0.0257)	(0.0375)	(0.0375)	(0.0377)
Mean of Dep. Var	29.1728	29.1728	29.1728	29.9229	29.9229	29.9229	27.3993	27.3993	27.3993
Mother and infant characteristics	No	Yes	Yes	No	Yes	Yes	No	Yes	Yes
Weather	No	No	Yes	No	No	Yes	No	No	Yes
DHS cluster FE	Yes	Yes	Yes	Yes	Yes	Yes	Yes	Yes	Yes
Birth year FE	Yes	Yes	Yes	Yes	Yes	Yes	Yes	Yes	Yes
2-degree grid cell-birth month FE	Yes	Yes	Yes	Yes	Yes	Yes	Yes	Yes	Yes
Observations	880992	880992	880992	624705	624705	624705	252525	252525	252525

Note: This table presents the regression results examining the impact of in utero extreme heat exposure on neonatal mortality in African countries. Each cell in the table represents a separate regression. Columns (1)–(3) show results for the full sample, while columns (4)–(6) focus on the rural sample, and columns (7)–(9) on the urban sample. The outcome variable, neonatal mortality, is equal to 1,000 if an infant dies before reaching 1

month of age, and 0 otherwise. The explanatory variables include Cumulative Heat (75) (Row 1), Cumulative Heat (85) (Row 2), Cumulative Heat (90) (Row 3), Cumulative Heat (95) (Row 4), Heat Days (75) (Row 5), Heat Days (85) (Row 6), Heat Days (90) (Row 7), and Heat Days (95) (Row 8). Controls for maternal and infant characteristics include the mother's age (and age squared) at birth, maternal education, infant gender, and birth order. We also control for monthly average precipitation in the DHS cluster during the 9-month pregnancy period, as well as DHS cluster, birth year, and 2-degree grid cell-birth month fixed effects in all specifications. Standard errors, clustered at the DHS cluster level, are reported in parentheses. Variable definitions are provided in SI Table A1. *, **, and *** indicate significance at the 10%, 5%, and 1% levels, respectively.

Table S7. Impact of in utero extreme heat exposure on neonatal mortality, excluding Eswatini

Dependent variable	Neonatal mortality		
	(1)	(2)	(3)
Cumulative heat (75)	0.0197***	0.0199***	0.0184***
	(0.0059)	(0.0059)	(0.0060)
Cumulative heat (85)	0.0271***	0.0274***	0.0253***
	(0.0085)	(0.0085)	(0.0086)
Cumulative heat (90)	0.0351***	0.0353***	0.0328***
	(0.0112)	(0.0112)	(0.0113)
Cumulative heat (95)	0.0554***	0.0555***	0.0517***
	(0.0175)	(0.0175)	(0.0176)
Heat days (75)	0.0358***	0.0360***	0.0330***
	(0.0122)	(0.0122)	(0.0123)
Heat days (85)	0.0447***	0.0449***	0.0414***
	(0.0147)	(0.0147)	(0.0149)
Heat days (90)	0.0539***	0.0540***	0.0498***
	(0.0178)	(0.0178)	(0.0180)
Heat days (95)	0.0934***	0.0929***	0.0873***
	(0.0255)	(0.0254)	(0.0257)
Mean of Dep. Var	29.9203	29.9203	29.9203
Mother and infant characteristics	No	Yes	Yes
Weather	No	No	Yes
DHS cluster FE	Yes	Yes	Yes
Birth year FE	Yes	Yes	Yes
2-degree grid cell-birth month FE	Yes	Yes	Yes
Observations	624693	624693	624693

Note: This table presents the robustness test results after excluding observations from Eswatini, based on the rural sample. Each cell in the table represents a separate regression. The outcome variable, neonatal mortality, equals 1,000 if an infant dies before reaching 1 month of age, and 0 otherwise. The explanatory variables include Cumulative Heat (75) (Row 1), Cumulative Heat (85) (Row 2), Cumulative Heat (90) (Row 3), Cumulative Heat (95) (Row 4), Heat Days (75) (Row 5), Heat Days (85) (Row 6), Heat Days (90) (Row 7), and Heat Days (95) (Row 8). The regressions control for monthly average precipitation in the DHS cluster during the 9-month pregnancy period, infant gender, birth order, mother's age (and age squared) at birth, maternal education, DHS cluster, and birth year fixed effects. Standard errors, clustered at the DHS cluster level, are reported in parentheses. Additionally, country-birth month fixed effects or 1-degree grid cell-birth month fixed effects are incorporated into the regressions in columns (1) and (2), respectively. Variable definitions are provided in SI Table A1. *, **, and *** indicate significance at the 10%, 5%, and 1% levels, respectively.

Table S8. Impact of in utero extreme heat exposure on neonatal mortality, alternative FE

Dependent variable	Neonatal mortality	
	(1)	(2)
Cumulative heat (75)	0.0141**	0.0152***
	(0.0056)	(0.0058)
Cumulative heat (85)	0.0207**	0.0229***
	(0.0082)	(0.0083)
Cumulative heat (90)	0.0271**	0.0302***
	(0.0109)	(0.0110)
Cumulative heat (95)	0.0440***	0.0487***
	(0.0170)	(0.0170)
Heat days (75)	0.0238**	0.0251**
	(0.0117)	(0.0119)
Heat days (85)	0.0321**	0.0341**
	(0.0142)	(0.0143)
Heat days (90)	0.0395**	0.0421**
	(0.0172)	(0.0173)
Heat days (95)	0.0752***	0.0801***
	(0.0248)	(0.0249)
Controls	Yes	Yes
DHS cluster FE	Yes	Yes
Birth year FE	Yes	Yes
Region-birth month FE	Country-birth month	1-degree grid cell -birth month
Observations	626934	626872

Note: This table presents the robustness test results for the rural sample, using different fixed effects. Each cell in the table represents a separate regression. The outcome variable, neonatal mortality, equals 1,000 if an infant dies before reaching 1 month of age, and 0 otherwise. The explanatory variables include Cumulative Heat (75) (Row 1), Cumulative Heat (85) (Row 2), Cumulative Heat (90) (Row 3), Cumulative Heat (95) (Row 4), Heat Days (75) (Row 5), Heat Days (85) (Row 6), Heat Days (90) (Row 7), and Heat Days (95) (Row 8). The regressions control for monthly average precipitation in the DHS cluster during the 9-month pregnancy period, infant gender, birth order, mother's age (and age squared) at birth, maternal education, DHS cluster, and birth year fixed effects in all specifications. Standard errors, clustered at the DHS cluster level, are reported in parentheses. Additionally, country-birth month fixed effects or 1-degree grid cell-birth month fixed effects are incorporated into the regressions presented in columns (1) and (2), respectively. Variable definitions are provided in SI Table A1. *, **, and *** indicate significance at the 10%, 5%, and 1% levels, respectively.

Table S9. Impact of in utero extreme heat exposure on neonatal mortality, accounting for migration

Dependent variable	Neonatal mortality		
	(1)	(2)	(3)
Cumulative heat (75)	0.0211***	0.0212***	0.0200***
	(0.0066)	(0.0066)	(0.0067)
Cumulative heat (85)	0.0289***	0.0291***	0.0275***
	(0.0096)	(0.0096)	(0.0097)
Cumulative heat (90)	0.0373***	0.0376***	0.0355***
	(0.0126)	(0.0126)	(0.0127)
Cumulative heat (95)	0.0539***	0.0541***	0.0511***
	(0.0195)	(0.0195)	(0.0197)
Heat days (75)	0.0441***	0.0444***	0.0419***
	(0.0139)	(0.0138)	(0.0140)
Heat days (85)	0.0561***	0.0563***	0.0534***
	(0.0167)	(0.0167)	(0.0169)
Heat days (90)	0.0641***	0.0640***	0.0605***
	(0.0201)	(0.0201)	(0.0203)
Heat days (95)	0.0999***	0.0999***	0.0953***
	(0.0288)	(0.0288)	(0.0291)
Mean of Dep. Var	30.1283	30.1283	30.1283
Mother and infant characteristics	No	Yes	Yes
Weather	No	No	Yes
DHS cluster FE	Yes	Yes	Yes
Birth year FE	Yes	Yes	Yes
2-degree grid cell-birth month FE	Yes	Yes	Yes
Observations	495747	495747	495747

Note: This table presents the robustness test results for the rural sample, restricted to mothers who spent their entire gestational period in their current residence. Each cell in the table represents a separate regression. The outcome variable, neonatal mortality, equals 1,000 if an infant dies before reaching 1 month of age, and 0 otherwise. The explanatory variables include Cumulative Heat (75) (Row 1), Cumulative Heat (85) (Row 2), Cumulative Heat (90) (Row 3), Cumulative Heat (95) (Row 4), Heat Days (75) (Row 5), Heat Days (85) (Row 6), Heat Days (90) (Row 7), and Heat Days (95) (Row 8). Controls for maternal and infant characteristics include the mother's age (and age squared) at birth, maternal education, infant gender, and birth order. We also controlled for monthly average precipitation in the DHS cluster during the 9-month pregnancy period, as well as DHS cluster, birth year, and 2-degree grid cell-birth month fixed effects in all specifications. Standard errors, clustered at the DHS cluster level, are reported in parentheses. Variable definitions are provided in SI Table A1. *, **, and *** indicate significance at the 10%, 5%, and 1% levels, respectively.

Table S10. Impact of in utero extreme heat exposure on neonatal mortality, accounting for postnatal exposure

Dependent variable	Neonatal mortality			
	Panel A			
	(1)	(2)	(3)	(4)
Cumulative heat (75)	0.0169***			
	(0.0060)			
Cumulative heat post (75)	0.0325			
	(0.0245)			
Cumulative heat (85)		0.0230***		
		(0.0086)		
Cumulative heat post (85)		0.0496		
		(0.0352)		
Cumulative heat (90)			0.0296***	
			(0.0114)	
Cumulative heat post (90)			0.0641	
			(0.0462)	
Cumulative heat (95)				0.0473***
				(0.0177)
Cumulative heat post (95)				0.0770
				(0.0720)
Controls	Yes	Yes	Yes	Yes
DHS cluster FE	Yes	Yes	Yes	Yes
Birth year FE	Yes	Yes	Yes	Yes
2-degree grid cell-birth month FE	Yes	Yes	Yes	Yes
Adj.R2	0.0087	0.0087	0.0087	0.0087
Observations	624705	624705	624705	624705
	Panel B			
	(1)	(2)	(3)	(4)
Heat days (75)	0.0317**			
	(0.0124)			
Heat days post (75)	0.0055			
	(0.0543)			
Heat days (85)		0.0373**		
		(0.0151)		
Heat days post (85)		0.0747		
		(0.0650)		
Heat days (90)			0.0441**	
			(0.0181)	
Heat days post (90)			0.1071	
			(0.0778)	
Heat days (95)				0.0796***
				(0.0260)
Heat days post (95)				0.1355
				(0.1081)
Controls	Yes	Yes	Yes	Yes
DHS cluster FE	Yes	Yes	Yes	Yes
Birth year FE	Yes	Yes	Yes	Yes
2-degree grid cell-birth month FE	Yes	Yes	Yes	Yes

Adj.R2	0.0087	0.0087	0.0087	0.0087
Observations	624705	624705	624705	624705

Note: This table presents the robustness test results based on the rural sample, controlling for heat exposure during the month of birth. Each column in the table represents a separate regression. The outcome variable, neonatal mortality, equals 1,000 if an infant dies before reaching 1 month of age, and 0 otherwise. The explanatory variables include Cumulative Heat (75), Cumulative Heat (85), Cumulative Heat (90), and Cumulative Heat (95) in Panel A, and Heat Days (75), Heat Days (85), Heat Days (90), and Heat Days (95) in Panel B, as well as corresponding heat exposure during the month of birth. The regressions control for monthly average precipitation in the DHS cluster during the 9-month pregnancy period, average precipitation in the birth month, infant gender, birth order, mother's age (and age squared) at birth, maternal education, as well as DHS cluster, birth year, and 2-degree grid cell-birth month fixed effects in all specifications. Standard errors, clustered at the DHS cluster level, are reported in parentheses. Variable definitions are provided in SI Table A1. *, **, and *** indicate significance at the 10%, 5%, and 1% levels, respectively.

Table S11. Impact of in utero extreme heat exposure on neonatal mortality, extreme cold exposure

Dependent variable	Neonatal mortality		
	(1)	(2)	(3)
Cumulative cold (75)	-0.0014	-0.0013	0.0001
	(0.0057)	(0.0057)	(0.0057)
Cumulative cold (85)	0.0012	0.0014	0.0032
	(0.0082)	(0.0082)	(0.0082)
Cumulative cold (90)	0.0014	0.0017	0.0039
	(0.0108)	(0.0108)	(0.0109)
Cumulative cold (95)	0.0045	0.0054	0.0082
	(0.0167)	(0.0167)	(0.0168)
Cold days (75)	-0.0182	-0.0187	-0.0151
	(0.0123)	(0.0123)	(0.0124)
Cold days (85)	-0.0086	-0.0093	-0.0051
	(0.0150)	(0.0149)	(0.0151)
Cold days (90)	-0.0083	-0.0090	-0.0043
	(0.0179)	(0.0179)	(0.0180)
Cold days (95)	-0.0069	-0.0077	-0.0020
	(0.0250)	(0.0249)	(0.0251)
Mean of Dep. Var	29.9229	29.9229	29.9229
Mother and infant characteristics	No	Yes	Yes
Weather	No	No	Yes
DHS cluster FE	Yes	Yes	Yes
Birth year FE	Yes	Yes	Yes
2-degree grid cell-birth month FE	Yes	Yes	Yes
Observations	624705	624705	624705

Note: This table presents the robustness test results for the impact of in utero extreme cold exposure based on the rural sample. Each cell in the table represents a separate regression. The outcome variable, neonatal mortality, equals 1,000 if an infant dies before reaching 1 month of age, and 0 otherwise. The explanatory variables include Cumulative Cold (25), Cumulative Cold (15), Cumulative Cold (10), Cumulative Cold (5), Cold Days (25), Cold Days (15), Cold Days (10), and Cold Days (5). The regressions control for monthly average precipitation in the DHS cluster during the 9-month pregnancy period, infant gender, birth order, mother's age (and age squared) at birth, maternal education, as well as DHS cluster, birth year, and 2-degree grid cell-birth month fixed effects in all specifications. Standard errors, clustered at the DHS cluster level, are reported in parentheses. Variable definitions are provided in SI Table A1. *, **, and *** indicate significance at the 10%, 5%, and 1% levels, respectively.

Table S12. Impact of in utero extreme heat exposure on neonatal mortality, 5 year window

Dependent variable	Neonatal mortality		
	(1)	(2)	(3)
Cumulative heat (75)	0.0204***	0.0205***	0.0166**
	(0.0073)	(0.0073)	(0.0074)
Cumulative heat (85)	0.0288***	0.0289***	0.0236**
	(0.0106)	(0.0106)	(0.0108)
Cumulative heat (90)	0.0373***	0.0373***	0.0305**
	(0.0141)	(0.0141)	(0.0143)
Cumulative heat (95)	0.0590***	0.0584***	0.0485**
	(0.0221)	(0.0221)	(0.0223)
Heat days (75)	0.0467***	0.0467***	0.0394**
	(0.0151)	(0.0151)	(0.0154)
Heat days (85)	0.0527***	0.0527***	0.0440**
	(0.0182)	(0.0182)	(0.0185)
Heat days (90)	0.0640***	0.0637***	0.0534**
	(0.0220)	(0.0220)	(0.0223)
Heat days (95)	0.1068***	0.1056***	0.0914***
	(0.0317)	(0.0317)	(0.0320)
Mean of Dep. Var	29.3754	29.3754	29.3754
Mother and infant characteristics	No	Yes	Yes
Weather	No	No	Yes
DHS cluster FE	Yes	Yes	Yes
Birth year FE	Yes	Yes	Yes
2-degree grid cell-birth month FE	Yes	Yes	Yes
Observations	453304	453304	453304

Note: This table presents the robustness test results after changing the extreme temperature benchmark, based on the rural sample. The benchmark temperature for calculating cumulative heat is set at the 75th, 85th, 90th, and 95th percentiles of the past 5 years' temperatures for the same season. Each cell in the table represents a separate regression. The outcome variable, neonatal mortality, equals 1,000 if an infant dies before reaching 1 month of age, and 0 otherwise. The explanatory variables include Cumulative Heat (75) (Row 1), Cumulative Heat (85) (Row 2), Cumulative Heat (90) (Row 3), and Cumulative Heat (95) (Row 4), as well as Heat Days (75) (Row 5), Heat Days (85) (Row 6), Heat Days (90) (Row 7), and Heat Days (95) (Row 8). Controls for maternal and infant characteristics include the mother's age (and age squared) at birth, maternal education, infant gender, and birth order. We also control for monthly average precipitation in the DHS cluster during the 9-month pregnancy period, as well as DHS cluster, birth year, and 2-degree grid cell region-birth month fixed effects in all specifications. Standard errors, clustered at the DHS cluster level, are reported in parentheses. Variable definitions are provided in SI Table A1. *, **, and *** indicate significance at the 10%, 5%, and 1% levels, respectively.

Table S13. Impact of in utero extreme heat exposure on neonatal mortality, 10 year window

Dependent variable	Neonatal mortality								
	Full sample			Rural sample			Urban sample		
	(1)	(2)	(3)	(4)	(5)	(6)	(7)	(8)	(9)
Cumulative heat (75)	0.0156***	0.0162***	0.0142**	0.0211***	0.0218***	0.0201***	0.0053	0.0060	0.0033
	(0.0058)	(0.0058)	(0.0059)	(0.0071)	(0.0071)	(0.0072)	(0.0105)	(0.0105)	(0.0106)
Cumulative heat (85)	0.0219**	0.0229***	0.0200**	0.0287***	0.0297***	0.0271**	0.0110	0.0121	0.0085
	(0.0087)	(0.0087)	(0.0088)	(0.0106)	(0.0106)	(0.0107)	(0.0161)	(0.0161)	(0.0162)
Cumulative heat (90)	0.0296**	0.0311**	0.0273**	0.0368**	0.0384***	0.0350**	0.0192	0.0208	0.0161
	(0.0121)	(0.0121)	(0.0122)	(0.0145)	(0.0145)	(0.0147)	(0.0226)	(0.0226)	(0.0227)
Cumulative heat (95)	0.0451**	0.0475**	0.0416**	0.0538**	0.0565**	0.0511**	0.0350	0.0380	0.0307
	(0.0207)	(0.0207)	(0.0208)	(0.0247)	(0.0246)	(0.0248)	(0.0398)	(0.0398)	(0.0400)
Heat days (75)	0.0249**	0.0258**	0.0212*	0.0404***	0.0415***	0.0376**	-0.0007	0.0001	-0.0062
	(0.0119)	(0.0119)	(0.0120)	(0.0147)	(0.0147)	(0.0149)	(0.0212)	(0.0212)	(0.0214)
Heat days (85)	0.0339**	0.0353**	0.0299**	0.0481***	0.0495***	0.0448**	0.0121	0.0136	0.0067
	(0.0150)	(0.0150)	(0.0152)	(0.0185)	(0.0184)	(0.0187)	(0.0267)	(0.0267)	(0.0270)
Heat days (90)	0.0516***	0.0533***	0.0471**	0.0696***	0.0711***	0.0656***	0.0283	0.0306	0.0229
	(0.0187)	(0.0187)	(0.0189)	(0.0230)	(0.0229)	(0.0232)	(0.0335)	(0.0335)	(0.0338)
Heat days (95)	0.0856***	0.0889***	0.0801***	0.1085***	0.1117***	0.1037***	0.0530	0.0566	0.0462
	(0.0291)	(0.0291)	(0.0294)	(0.0355)	(0.0355)	(0.0359)	(0.0532)	(0.0531)	(0.0535)
Mean of Dep. Var	29.1728	29.1728	29.1728	29.9229	29.9229	29.9229	27.3993	27.3993	27.3993
Mother and infant characteristics	No	Yes	Yes	No	Yes	Yes	No	Yes	Yes
Weather	No	No	Yes	No	No	Yes	No	No	Yes
DHS cluster FE	Yes	Yes	Yes	Yes	Yes	Yes	Yes	Yes	Yes
Birth year FE	Yes	Yes	Yes	Yes	Yes	Yes	Yes	Yes	Yes
2-degree grid cell-birth month FE	Yes	Yes	Yes	Yes	Yes	Yes	Yes	Yes	Yes
Observations	880992	880992	880992	624705	624705	624705	252525	252525	252525

Note: This table presents regression results examining the impact of in utero exposure to extreme heat on neonatal mortality in African countries. We use four percentile thresholds of seasonal wet-bulb temperature (from 2003 to 2020) as benchmarks to calculate extreme heat exposure, after obtaining DHS cluster-specific distributions of temperature using a 10-year window. Each cell in the table corresponds to a separate regression. Columns (1)–(3) report results for the full sample, columns (4)–(6) for the rural subsample, and columns (7)–(9) for the urban subsample. The outcome variable, neonatal mortality, is coded as 1,000 if an infant dies within the first month of life and 0 otherwise. Key explanatory variables include Cumulative Heat based on the 75th, 85th, 90th, and 95th percentiles (Rows 1–4), and Heat Days defined by the same thresholds (Rows 5–8). Control variables include maternal and infant characteristics—mother's age (and age squared) at birth, maternal education, infant gender, and birth order—as well as monthly average precipitation in the DHS cluster during the 9-month pregnancy period. All regressions include DHS cluster fixed effects, birth year fixed effects, and 2-degree grid cell-by-birth-month fixed effects. Standard errors, clustered at the DHS cluster level, are reported in parentheses. Variable definitions are provided in SI Table A1. *, **, and *** denote statistical significance at the 10%, 5%, and 1% levels, respectively.

Table S14. Impact of in utero extreme heat exposure on neonatal mortality, 18 year window

Dependent variable	Neonatal mortality								
	Full sample			Rural sample			Urban sample		
	(1)	(2)	(3)	(4)	(5)	(6)	(7)	(8)	(9)
Cumulative heat (75)	0.0158***	0.0164***	0.0144**	0.0222***	0.0229***	0.0211***	0.0047	0.0053	0.0027
	(0.0058)	(0.0058)	(0.0059)	(0.0072)	(0.0072)	(0.0073)	(0.0105)	(0.0105)	(0.0106)
Cumulative heat (85)	0.0222**	0.0231***	0.0202**	0.0305***	0.0315***	0.0289***	0.0094	0.0104	0.0070
	(0.0087)	(0.0087)	(0.0088)	(0.0107)	(0.0107)	(0.0108)	(0.0158)	(0.0158)	(0.0159)
Cumulative heat (90)	0.0300**	0.0313***	0.0276**	0.0403***	0.0417***	0.0384***	0.0140	0.0155	0.0111
	(0.0119)	(0.0119)	(0.0120)	(0.0145)	(0.0145)	(0.0147)	(0.0217)	(0.0216)	(0.0218)
Cumulative heat (95)	0.0467**	0.0488**	0.0433**	0.0611**	0.0635***	0.0585**	0.0224	0.0249	0.0187
	(0.0197)	(0.0197)	(0.0199)	(0.0239)	(0.0239)	(0.0241)	(0.0366)	(0.0366)	(0.0368)
Heat days (75)	0.0241**	0.0249**	0.0202*	0.0409***	0.0419***	0.0379**	-0.0015	-0.0007	-0.0071
	(0.0118)	(0.0118)	(0.0120)	(0.0146)	(0.0146)	(0.0148)	(0.0213)	(0.0212)	(0.0215)
Heat days (85)	0.0269*	0.0282*	0.0225	0.0431**	0.0443**	0.0394**	0.0027	0.0043	-0.0027
	(0.0148)	(0.0148)	(0.0150)	(0.0183)	(0.0183)	(0.0185)	(0.0266)	(0.0265)	(0.0268)
Heat days (90)	0.0414**	0.0430**	0.0366**	0.0616***	0.0631***	0.0573**	0.0143	0.0165	0.0089
	(0.0184)	(0.0184)	(0.0186)	(0.0227)	(0.0227)	(0.0230)	(0.0327)	(0.0327)	(0.0330)
Heat days (95)	0.0859***	0.0889***	0.0803***	0.1162***	0.1191***	0.1112***	0.0401	0.0437	0.0341
	(0.0280)	(0.0280)	(0.0283)	(0.0347)	(0.0347)	(0.0351)	(0.0496)	(0.0496)	(0.0499)
Mean of Dep. Var	29.1728	29.1728	29.1728	29.9229	29.9229	29.9229	27.3993	27.3993	27.3993
Mother and infant characteristics	No	Yes	Yes	No	Yes	Yes	No	Yes	Yes
Weather	No	No	Yes	No	No	Yes	No	No	Yes
DHS cluster FE	Yes	Yes	Yes	Yes	Yes	Yes	Yes	Yes	Yes
Birth year FE	Yes	Yes	Yes	Yes	Yes	Yes	Yes	Yes	Yes
2-degree grid cell-birth month FE	Yes	Yes	Yes	Yes	Yes	Yes	Yes	Yes	Yes
Observations	880992	880992	880992	624705	624705	624705	252525	252525	252525

Note: This table presents regression results examining the impact of in utero exposure to extreme heat on neonatal mortality in African countries. We use four percentile thresholds of seasonal wet-bulb temperature (from 2003 to 2020) as benchmarks to calculate extreme heat exposure, after obtaining DHS cluster-specific distributions of temperature using an 18-year window. Each cell in the table corresponds to a separate regression.

Columns (1)–(3) report results for the full sample, columns (4)–(6) for the rural subsample, and columns (7)–(9) for the urban subsample. The outcome variable, neonatal mortality, is coded as 1,000 if an infant dies within the first month of life and 0 otherwise. Key explanatory variables include Cumulative Heat based on the 75th, 85th, 90th, and 95th percentiles (Rows 1–4), and Heat Days defined by the same thresholds (Rows 5–8). Control variables include maternal and infant characteristics—mother's age (and age squared) at birth, maternal education, infant gender, and birth order—as well as monthly average precipitation in the DHS cluster during the 9-month pregnancy period. All regressions include DHS cluster fixed effects, birth year fixed effects, and 2-degree grid cell-by-birth-month fixed effects. Standard errors, clustered at the DHS cluster level, are reported in parentheses. Variable definitions are provided in SI Table A1. *, **, and *** denote statistical significance at the 10%, 5%, and 1% levels, respectively.

Table S15. Impact of in utero extreme heat exposure on prenatal checks

Dependent variable	No. of prenatal checks			
	Panel A			
	(1)	(2)	(3)	(4)
Cumulative heat (75)	-0.0003**			
	(0.0001)			
Cumulative heat (85)		-0.0006***		
		(0.0002)		
Cumulative heat (90)			-0.0007***	
			(0.0003)	
Cumulative heat (95)				-0.0014***
				(0.0004)
Mean of Dep. Var	3.6825	3.6825	3.6825	3.6825
Controls	Yes	Yes	Yes	Yes
DHS cluster FE	Yes	Yes	Yes	Yes
Birth year FE	Yes	Yes	Yes	Yes
2-degree grid cell-birth month FE	Yes	Yes	Yes	Yes
Adj.R2	0.4440	0.4440	0.4440	0.4440
Observations	269657	269657	269657	269657
	Panel B			
	(1)	(2)	(3)	(4)
Heat days (75)	-0.0006**			
	(0.0003)			
Heat days (85)		-0.0009**		
		(0.0004)		
Heat days (90)			-0.0010**	
			(0.0004)	
Heat days (95)				-0.0020***
				(0.0006)
Mean of Dep. Var	3.6825	3.6825	3.6825	3.6825
Controls	Yes	Yes	Yes	Yes
DHS cluster FE	Yes	Yes	Yes	Yes
Birth year FE	Yes	Yes	Yes	Yes
2-degree grid cell-birth month FE	Yes	Yes	Yes	Yes
Adj.R2	0.4440	0.4440	0.4440	0.4440
Observations	269657	269657	269657	269657

Note: This table presents the regression results examining the impact of in utero extreme heat exposure on the frequency of prenatal checks among rural pregnant women in African countries. Each column in the table represents a separate regression. The outcome variable is the number of hospital visits a mother makes for medical care during pregnancy (No. of prenatal checks). The explanatory variables include Cumulative Heat (75) (Column 1), Cumulative Heat (85) (Column 2), Cumulative Heat (90) (Column 3), and Cumulative Heat (95) (Column 4) in Panel A, and Heat Days (75) (Column 1), Heat Days (85) (Column 2), Heat Days (90) (Column 3), and Heat Days (95) (Column 4) in Panel B. The regressions control for monthly average precipitation in the DHS cluster during the 9-month pregnancy period, birth order, mother's age (and age squared) at birth, maternal education, DHS cluster, birth year, and 2-degree grid cell-birth month fixed effects. Standard errors, clustered at the DHS cluster level, are reported in parentheses. Variable definitions are provided in SI Table A1. *, **, and *** indicate significance at the 10%, 5%, and 1% levels, respectively.

Table S16. Impact of in utero extreme heat exposure on neonatal mortality, by source of drinking water and sanitation

Dependent variable	Neonatal mortality			
	Source of drinking water		Type of sanitation facility	
	(1)	(2)	(3)	(4)
	Unimproved	Improved	Unimproved	Improved
Cumulative heat (75)	0.0256**	0.0167**	0.0241***	0.0148
	(0.0102)	(0.0078)	(0.0077)	(0.0104)
Cumulative heat (85)	0.0396***	0.0198*	0.0363***	0.0131
	(0.0146)	(0.0112)	(0.0111)	(0.0150)
Cumulative heat (90)	0.0517***	0.0250*	0.0467***	0.0156
	(0.0192)	(0.0147)	(0.0147)	(0.0194)
Cumulative heat (95)	0.0764**	0.0407*	0.0803***	0.0098
	(0.0298)	(0.0228)	(0.0230)	(0.0294)
Heat days (75)	0.0385*	0.0360**	0.0371**	0.0352
	(0.0216)	(0.0157)	(0.0159)	(0.0214)
Heat days (85)	0.0528**	0.0388**	0.0523***	0.0354
	(0.0262)	(0.0191)	(0.0192)	(0.0259)
Heat days (90)	0.0752**	0.0421*	0.0647***	0.0328
	(0.0314)	(0.0230)	(0.0233)	(0.0308)
Heat days (95)	0.1072**	0.0848**	0.1102***	0.0594
	(0.0446)	(0.0330)	(0.0334)	(0.0436)
Mean of Dep. Var	31.2864	29.2078	30.5532	28.8764
Controls	Yes	Yes	Yes	Yes
DHS cluster FE	Yes	Yes	Yes	Yes
Birth year FE	Yes	Yes	Yes	Yes
2-degree grid cell-birth month FE	Yes	Yes	Yes	Yes
Observations	236109	380823	402805	213323

Note: This table presents the heterogeneous effects of in utero extreme heat exposure on neonatal mortality by source of drinking water and type of sanitation in rural areas. Each cell in the table represents a separate regression. The outcome variable, neonatal mortality, equals 1,000 if an infant dies before reaching 1 month of age, and 0 otherwise. The explanatory variables include Cumulative Heat (75) (Row 1), Cumulative Heat (85) (Row 2), Cumulative Heat (90) (Row 3), Cumulative Heat (95) (Row 4), Heat Days (75) (Row 5), Heat Days (85) (Row 6), Heat Days (90) (Row 7), and Heat Days (95) (Row 8). In columns (1) and (2), we separately examine the impacts on households using unimproved and improved sources of water, respectively. In columns (3) and (4), we explore the heterogeneous effects on households using unimproved and improved sanitation facilities, respectively. The regressions control for monthly average precipitation in the DHS cluster during the 9-month pregnancy period, infant gender, birth order, mother's age (and age squared) at birth, maternal education, as well as DHS cluster, birth year, and 2-degree grid cell-birth month fixed effects. Standard errors, clustered at the DHS cluster level, are reported in parentheses. Variable definitions are provided in SI Table A1. *, **, and *** indicate significance at the 10%, 5%, and 1% levels, respectively.

Table S17. Impact of in utero extreme heat exposure on neonatal mortality, by household wealth, access to electricity, and mother's education level

Dependent variable	Neonatal mortality					
	Household wealth		Electricity		Mother's education	
	(1)	(2)	(3)	(4)	(5)	(6)
	Poor/middle	Rich	No	Yes	No/primary	Secondary/higher
Cumulative heat (75)	0.0186***	0.0105	0.0198***	0.0104	0.0181***	0.0211
	(0.0066)	(0.0160)	(0.0067)	(0.0147)	(0.0065)	(0.0182)
Cumulative heat (85)	0.0259***	0.0112	0.0295***	-0.0011	0.0247***	0.0237
	(0.0096)	(0.0230)	(0.0096)	(0.0214)	(0.0094)	(0.0259)
Cumulative heat (90)	0.0337***	0.0145	0.0381***	-0.0064	0.0313**	0.0305
	(0.0126)	(0.0298)	(0.0126)	(0.0280)	(0.0124)	(0.0338)
Cumulative heat (95)	0.0549***	0.0148	0.0602***	-0.0207	0.0475**	0.0589
	(0.0195)	(0.0467)	(0.0196)	(0.0429)	(0.0193)	(0.0520)
Heat days (75)	0.0309**	0.0452	0.0297**	0.0484	0.0297**	0.0548
	(0.0137)	(0.0327)	(0.0139)	(0.0302)	(0.0135)	(0.0369)
Heat days (85)	0.0410**	0.0356	0.0442***	0.0262	0.0379**	0.0466
	(0.0166)	(0.0402)	(0.0168)	(0.0363)	(0.0163)	(0.0445)
Heat days (90)	0.0472**	0.0544	0.0571***	0.0087	0.0434**	0.0655
	(0.0200)	(0.0482)	(0.0202)	(0.0435)	(0.0196)	(0.0539)
Heat days (95)	0.0847***	0.0812	0.0958***	0.0252	0.0826***	0.0977
	(0.0285)	(0.0696)	(0.0288)	(0.0633)	(0.0280)	(0.0783)
Mean of Dep. Var	30.3632	28.2259	30.2213	28.8891	30.4603	27.6215
Mother and infant characteristics	Yes	Yes	Yes	Yes	Yes	Yes
Weather	Yes	Yes	Yes	Yes	Yes	Yes
DHS cluster FE	Yes	Yes	Yes	Yes	Yes	Yes
Birth year FE	Yes	Yes	Yes	Yes	Yes	Yes
2-degree grid cell-birth month FE	Yes	Yes	Yes	Yes	Yes	Yes
Observations	517732	97499	507357	110526	530330	82653

Note: This table presents the heterogeneous effects of in utero extreme heat exposure on neonatal mortality by household wealth, access to electricity, and mother's education level. Each cell in the table represents a separate regression. The outcome variable, neonatal mortality, equals 1,000 if an infant dies before reaching 1 month of age, and 0 otherwise. The explanatory variables include Cumulative Heat (75) (Row 1), Cumulative Heat (85) (Row 2), Cumulative Heat (90) (Row 3), Cumulative Heat (95) (Row 4), Heat Days (75) (Row 5), Heat Days (85) (Row 6), Heat Days (90) (Row 7), and Heat Days (95) (Row 8). We explore the heterogeneous effects on households by wealth categories, including poor (poorest and poor) and middle-class in column (1), and rich (richer and richest) in column (2). In columns (3) and (4), we separate the sample based on access to electricity, while in columns (5) and (6), we examine the effects based on the mother's education level. The regressions control for monthly average precipitation in the DHS cluster during the 9-month pregnancy period, infant gender, birth order, mother's age (and age squared) at birth, maternal education, as well as DHS cluster, birth year, and 2-degree grid cell-birth month fixed effects. Standard errors, clustered at the DHS cluster level, are reported in parentheses. Variable definitions are provided in SI Table A1. *, **, and *** indicate significance at the 10%, 5%, and 1% levels, respectively.

Table S18. Impact of in utero extreme heat exposure on neonatal mortality, by employment

Dependent variable	Neonatal mortality	
	(1) Agriculture	(2) Others
Cumulative heat (75)	0.0148	0.0156**
	(0.0092)	(0.0064)
Cumulative heat (85)	0.0227*	0.0216**
	(0.0131)	(0.0092)
Cumulative heat (90)	0.0308*	0.0281**
	(0.0171)	(0.0121)
Cumulative heat (95)	0.0521**	0.0404**
	(0.0262)	(0.0190)
Heat days (75)	0.0247	0.0215
	(0.0186)	(0.0132)
Heat days (85)	0.0449**	0.0231
	(0.0225)	(0.0159)
Heat days (90)	0.0539**	0.0334*
	(0.0269)	(0.0192)
Heat days (95)	0.0834**	0.0742***
	(0.0379)	(0.0275)
Mean of Dep. Var	28.9760	29.3704
Mother and infant characteristics	Yes	Yes
Weather	Yes	Yes
DHS cluster FE	Yes	Yes
Birth year FE	Yes	Yes
2-degree grid cell-birth month FE	Yes	Yes
Observations	281095	541192

Note: This table presents the heterogeneous effects of in utero extreme heat exposure on neonatal mortality by respondents' occupation. We examine differences in impact based on whether the mother reports working in agriculture. Each cell in the table represents a separate regression. The outcome variable, neonatal mortality, equals 1,000 if an infant dies before reaching one month of age, and 0 otherwise. The explanatory variables include Cumulative Heat (75) (Row 1), Cumulative Heat (85) (Row 2), Cumulative Heat (90) (Row 3), Cumulative Heat (95) (Row 4), Heat Days (75) (Row 5), Heat Days (85) (Row 6), Heat Days (90) (Row 7), and Heat Days (95) (Row 8). The regressions control for monthly average precipitation in the DHS cluster during the 9-month pregnancy period, infant gender, birth order, mother's age (and age squared) at birth, and maternal education, as well as DHS cluster, birth year, and 2-degree grid cell–birth month fixed effects. Standard errors, clustered at the DHS cluster level, are reported in parentheses. Variable definitions are provided in SI Table A1. *, **, and *** indicate significance at the 10%, 5%, and 1% levels, respectively.

Table S19. In utero heat exposure and neonatal mortality: Access to professional delivery

Dependent variable	Neonatal mortality	
	(1)	(2)
	Without professional delivery	With professional delivery
Cumulative heat (75)	0.0247**	0.0115
	(0.0116)	(0.0086)
Cumulative heat (85)	0.0353**	0.0192
	(0.0168)	(0.0124)
Cumulative heat (90)	0.0479**	0.0281*
	(0.0221)	(0.0163)
Cumulative heat (95)	0.0657*	0.0516**
	(0.0351)	(0.0256)
Heat days (75)	0.0506**	0.0117
	(0.0243)	(0.0171)
Heat days (85)	0.0588**	0.0220
	(0.0297)	(0.0208)
Heat days (90)	0.0654*	0.0362
	(0.0358)	(0.0252)
Heat days (95)	0.1091**	0.0594*
	(0.0511)	(0.0358)
Mean of Dep. Var	29.8056	26.8684
Mother and infant characteristics	Yes	Yes
Weather	Yes	Yes
DHS cluster FE	Yes	Yes
Birth year FE	Yes	Yes
2-degree grid cell-birth month FE	Yes	Yes
Observations	212242	328229

Note: This table presents the heterogeneous effects of in utero exposure to extreme heat on neonatal mortality, differentiated by whether a professional assisted during delivery. Each cell reports the result from a separate regression. The outcome variable, neonatal mortality, is coded as 1,000 if an infant dies within the first month of life and 0 otherwise. The key explanatory variables include: Cumulative Heat (75th percentile) in Row 1, Cumulative Heat (85th) in Row 2, Cumulative Heat (90th) in Row 3, and Cumulative Heat (95th) in Row 4; followed by Heat Days (75th) in Row 5, Heat Days (85th) in Row 6, Heat Days (90th) in Row 7, and Heat Days (95th) in Row 8. All regressions control for monthly average precipitation in the DHS cluster during the 9-month pregnancy period, infant gender, birth order, mother's age (and age squared) at birth, and maternal education. In addition, we include DHS cluster fixed effects, birth year fixed effects, and 2-degree grid cell-by-birth-month fixed effects. Standard errors, clustered at the DHS cluster level, are reported in parentheses. Variable definitions are provided in SI Table A1. *, **, and *** indicate statistical significance at the 10%, 5%, and 1% levels, respectively.
